# Similarly Lethal Strains of Extraintestinal Pathogenic *Escherichia coli* Trigger Markedly Diverse Host Responses in a Zebrafish Model of Sepsis

**DOI:** 10.1128/mSphere.00062-16

**Published:** 2016-04-20

**Authors:** Amelia E. Barber, Brittany A. Fleming, Matthew A. Mulvey

**Affiliations:** Division of Microbiology and Immunology, Pathology Department, University of Utah, Salt Lake City, Utah, USA; University of Kentucky

**Keywords:** *Escherichia coli*, ExPEC, TLR5, bacteremia, bloodstream infections, cytokine storm, flagellin, sepsis, zebrafish

## Abstract

In individuals with sepsis, the infecting microbes are commonly viewed as generic inducers of inflammation while the host background is considered the primary variable affecting disease progression and outcome. To study the effects of bacterial strain differences on the maladaptive immune responses that are induced during sepsis, we employed a novel zebrafish embryo infection model using extraintestinal pathogenic *Escherichia coli* (ExPEC) isolates. These genetically diverse pathogens are a leading cause of sepsis and are becoming increasingly dangerous because of the rise of multidrug-resistant strains. Zebrafish infected with ExPEC isolates exhibit many of the pathophysiological features seen in septic human patients, including dysregulated inflammatory responses (cytokine storms), tachycardia, endothelial leakage, and progressive edema. However, only a limited subset of ExPEC isolates can trigger a sepsis-like state and death of the host when introduced into the bloodstream. Mirroring the situation in human patients, antibiotic therapy reduced ExPEC titers and improved host survival rates but was only effective within limited time frames that varied, depending on the infecting pathogen. Intriguingly, we find that phylogenetically distant but similarly lethal ExPEC isolates can stimulate markedly different host transcriptional responses, including disparate levels of inflammatory mediators. These differences correlate with the amounts of bacterial flagellin expression during infection, as well as differential activation of Toll-like receptor 5 by discrete flagellar serotypes. Altogether, this work establishes zebrafish as a relevant model of key aspects of human sepsis and highlights the ability of genetically distinct ExPEC isolates to induce divergent host responses independently of baseline host attributes.

**IMPORTANCE** Sepsis is a life-threatening systemic inflammatory condition that is initiated by the presence of microorganisms in the bloodstream. In the United States, sepsis due to ExPEC and other pathogens kills well over a quarter of a million people each year and is associated with tremendous health care costs. A high degree of heterogeneity in the signs and symptomology of sepsis makes this disease notoriously difficult to effectively diagnose and manage. Here, using a zebrafish model of sepsis, we find that similarly lethal but genetically distinct ExPEC isolates can elicit notably disparate host responses. These variances are in part due to differences in the levels and types of flagellin that are expressed by the infecting ExPEC strains. A better understanding of the variable impact that bacterial factors like flagellin have on host responses during sepsis could lead to improved diagnostic and therapeutic approaches to these often deadly infections.

**Podcast**: A podcast concerning this article is available.

## Abstract (precis)

Sepsis is a life-threatening systemic inflammatory condition that is initiated by the presence of microorganisms in the bloodstream. In the United States, sepsis due to ExPEC and other pathogens kills well over a quarter of a million people each year and is associated with tremendous health care costs. A high degree of heterogeneity in the signs and symptomology of sepsis makes this disease notoriously difficult to effectively diagnose and manage. Here, using a zebrafish model of sepsis, we find that similarly lethal but genetically distinct ExPEC isolates can elicit notably disparate host responses. These variances are in part due to differences in the levels and types of flagellin that are expressed by the infecting ExPEC strains. A better understanding of the variable impact that bacterial factors like flagellin have on host responses during sepsis could lead to improved diagnostic and therapeutic approaches to these often deadly infections.

## INTRODUCTION

*Escherichia coli* is an incredibly diverse species, both genetically and in terms of its ability to colonize numerous niches in the environment and within animal hosts. The relationship between *E. coli* and its animal hosts can be mutualistic, as is thought to be the case for most *E. coli* strains within the intestinal tracts of mammals, or pathogenic, causing diarrheal diseases, urinary tract infections, meningitis, sepsis, and other maladies. Strains that can instigate disease outside the intestinal tract, termed extraintestinal pathogenic *E. coli* (ExPEC), are very common and have a huge impact on human health and mortality (1). The ability of some ExPEC strains to gain access to and disseminate within the bloodstream is especially problematic and often lethal. ExPEC is the principal cause of bacteremia and a leading cause of sepsis, second only to group B *Streptococcus* in neonates and *Staphylococcus aureus* in adults (2–6). Over the past few decades, there has been a troubling increase in the rates of *E. coli*-induced sepsis in both adults and neonates, but the pathogenesis of these infections remains, for the most part, undetermined.

During sepsis, the generation of excessive inflammatory mediators, including cytokines and reactive oxygen species, can result in vascular leakage, disseminated intravascular coagulation (DIC), and organ failure. Clinically, sepsis patients may present with highly variable signs and symptoms that can make it difficult to assess disease severity or predict outcomes (7, 8). Much of the variability in disease progression in sepsis patients has been attributed to differences in the genetic background and immune status of individual hosts (9–11), but the nature of the infecting microbes can also impact disease outcome. For example, relative to Gram-positive pathogens and other Gram-negative bacteria, ExPEC is generally more inflammatory and more likely to cause death during sepsis (12, 13). Animal models indicate that the survival, growth, and virulence of specific ExPEC isolates within the bloodstream can be influenced by myriad bacterial genes, including those involved in adhesion, iron utilization, metabolism, membrane transport, toxin biosynthesis, and the production and modification of lipopolysaccharide and capsules (14–20). However, in epidemiological and genomics-based studies, no single set of bacterial genes has been identified that consistently correlates with the survival or lethality of ExPEC strains within the bloodstream of the human host (11, 21–26). These findings indicate that, as a group, ExPEC strains may utilize a diverse array of functionally redundant genes to deal with host defenses and other challenges during systemic infections.

An ExPEC isolate typically possesses about 5,000 genes, but specific gene content can differ between individual ExPEC strains by as much as 30% (27). The total number of distinct genes that may be shared among all ExPEC isolates and related *E. coli* strains in nature is referred to as the pangenome and is currently estimated to total more than 14,000 (21, 27, 28). Most of these genes are functionally undefined, often making it difficult to correlate bacterial virulence properties with gene function. The mosaic nature of ExPEC genomes helps explain previous observations showing that the lethality of even closely related isolates can vary markedly in animal models (24, 29, 30). It is likewise feasible that phylogenetically dissimilar ExPEC isolates can be equally lethal but have differential effects on host signaling pathways and inflammatory responses.

Here, we set out to define how different ExPEC isolates impact host responses during sepsis. We present a novel model of studying sepsis showing that inoculation of ExPEC into the bloodstream of zebrafish embryos can elicit many of the pathophysiological and transcriptional responses seen during human sepsis.

In this model, many of the ExPEC strains tested differ in virulence potential and even similarly lethal isolates were found to trigger notably divergent host responses. This phenomenon correlates with differences in the amounts and types of flagellin expressed by the lethal isolates and could, in part, account for the variability in the symptoms experienced by human sepsis patients.

## RESULTS

### A limited subset of *E. coli* strains causes host death following entry into the bloodstream.

**FIG 1:**
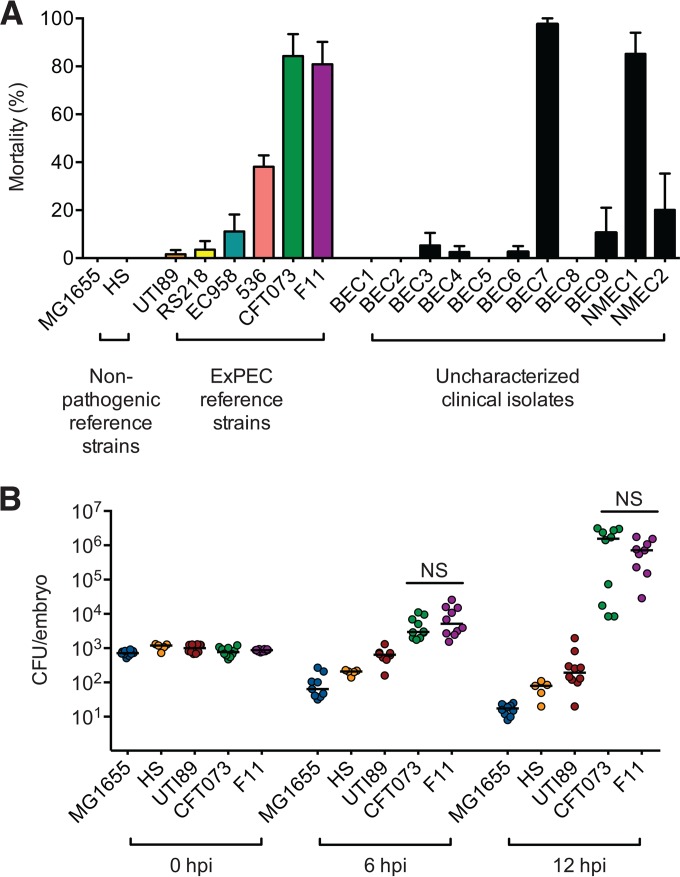
Few *E. coli* isolates can proliferate in the bloodstream and cause host death. (A) Lethality of nonpathogenic reference *E. coli* strains, reference ExPEC strains, and uncharacterized clinical isolates in zebrafish embryos 24 h following the injection of ~1,000 CFU into the bloodstream. Bars indicate the average percentages (± the standard error of the mean) of fish that were killed; *n* = 30 to 60 embryos pooled from two to four experiments. (B) Bacterial burdens in infected embryos at 0, 6, and 12 hpi. Data from two independent experiments were pooled. Lines mark median values; *n* = 5 to 10 embryos per time point. NS, no significant difference between CFT073 and F11 titers detected at any time point, as determined by Student’s *t* test.

To investigate the potential of different ExPEC isolates to induce various host responses during systemic infections, a panel of *E. coli* strains from various sources were injected into the bloodstreams of zebrafish embryos at 48 h postfertilization (hpf). At this developmental stage, the zebrafish immune system consists solely of innate defenses, including macrophages, neutrophils, complement, Toll-like receptors (TLRs), antimicrobial peptides, and cytokines similar to those encoded by humans (31, 32). Using an inoculation dose of approximately 700 to 1,500 CFU per embryo, we found that nonpathogenic K-12 strain MG1655 and the human gut isolate HS caused no death at 24 h postinoculation (hpi), whereas sequenced ExPEC reference strains F11 and CFT073 were both highly lethal within the same time frame (Fig. 1A). Two other, mostly uncharacterized, ExPEC isolates (BEC7 and NMEC1) were similarly lethal. The remaining ExPEC strains tested were, for the most part, nonfatal, with the exception of pyelonephritis isolate 536, which killed about 40% of the fish within 24 h. Of the *E. coli* strains tested, only about 26% killed more than 30% of the zebrafish following injection into the bloodstream.

In zebrafish, the lethality of an *E. coli* strain generally correlates with its ability to survive and multiply within the host (29). This trend holds true here. We found that the titers of nonlethal strains MG1655 and HS were greatly reduced within 12 hpi, while the numbers of nonlethal ExPEC isolate UTI89 bacteria remained relatively stable in the same time frame (Fig. 1B). In contrast, lethal reference ExPEC strains F11 and CFT073 not only persisted within the zebrafish host but also multiplied, on average, more than 1,000-fold by 12 hpi. Of note, all of the *E. coli* strains used here grew at similar rates in broth culture at 37°C and at 28.5°C, the temperature at which the zebrafish embryos are maintained (see Fig. S1 in the supplemental material). Taken together, these results highlight the strain-dependent lethality of *E. coli* following inoculation into the zebrafish bloodstream and are in line with previous observations indicating that the bloodstream is a highly restrictive environment for many bacteria (14, 29, 33).

10.1128/mSphere.00062-16.2 Figure S1  Similar growth rates of *E. coli* strains in broth culture. (A and B) Representative data showing that all of the strains tested show similar growth kinetics in LB broth at 28.5°C (A) and 37°C (B). Each graph is representative of three independent experiments performed in quadruplicate. Download Figure S1, PDF file, 0.2 MB. Copyright © 2016 Barber et al. 2016 Barber et al. This content is distributed under the terms of the Creative Commons Attribution 4.0 International license.

### Differential pathophysiological effects of similarly lethal but phylogenetically distant ExPEC isolates.

**FIG 2:**
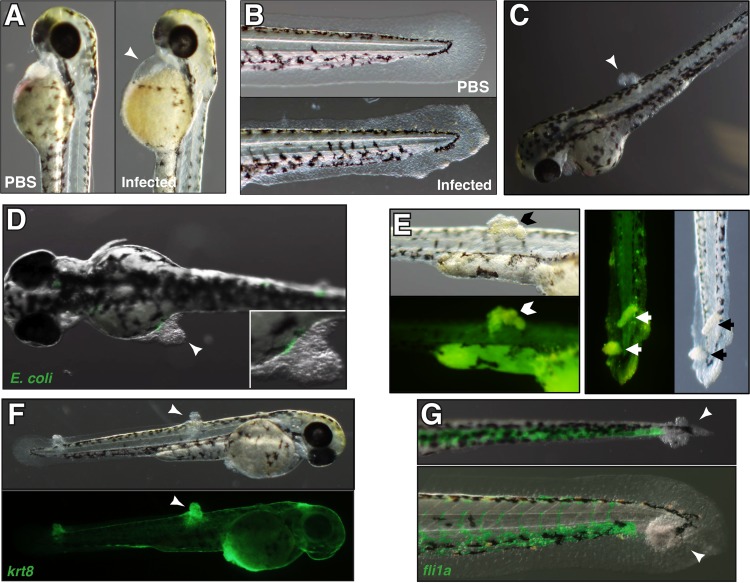
Distinct pathologies associated with different but equally lethal ExPEC isolates. (A) An F11-infected embryo displaying characteristic pericardial edema (arrowhead) at 12 hpi. This is not seen following the injection of controls with PBS (left). (B) A CFT073-infected embryo at 12 hpi showing fin erosion and ulceration commonly seen during infection with either CFT073 or F11. (C) F11-infected embryos, but not those infected with CFT073, often develop protrusions (arrowhead) in the trunk or tail region by 12 hpi. (D) Merged bright-field and fluorescent images of an F11/pGEN-GFP(LVA)-infected embryo at 12 hpi showing bacteria at the base of a protrusion (inset) but not within the main structure. (E) Bright-field and matched fluorescent images of F11-infected embryos stained with acridine orange (green) at 12 hpi. The dye accumulates within protrusions on the trunk (left, Chevron) and tail (right, arrows). (F) Bright-field (top) or fluorescent (bottom) images of Tg(*krt8*:GFP) embryos at 12 hpi with F11. Epithelial cells within this transgenic line express GFP. Each arrowhead marks an F11-induced protrusion. (G) Merged bright-field and fluorescent images of Tg(*fli1a*:GFP) embryos at 12 hpi with F11. Endothelial cells and leukocytes in this transgenic line express GFP. Protrusions are indicated by arrowheads.

F11 and CFT073 are phylogenetically distinct strains, as determined by multilocus sequence typing. Although they both encode the same K2 capsular and O6 surface antigens, they possess distinct flagellar serotypes, different genomic islands, and only partially overlapping sets of recognized virulence factors (28, 29, 34). F11 and CFT073 are about 73% identical at the protein coding level. Though these two strains are similarly lethal and grow at comparable rates within the zebrafish host, embryos infected with F11 appeared qualitatively more debilitated than embryos infected with CFT073. Infection with both strains led to overt signs of illness, including pericardial edema, ulceration of the skin, and erosion of the tail fin, compared to phosphate-buffered saline (PBS)-injected control fish (Fig. 2A and B). However, these phenotypes were markedly more pronounced and common in fish infected with F11.

Infection with F11 also resulted in the development of prominent tissue protrusions on the trunk or tail of the embryo (Fig. 2C). These protrusions were found on up to 50% of the fish infected with F11 but were not seen in CFT073-infected embryos. Infection with F11 carrying a plasmid encoding destabilized green fluorescent protein (GFP) [F11/pGEN-GFP(LVA)] revealed that the protrusions themselves did not contain bacteria, but small bacterial clusters were often detected within the tissues and vessels adjacent to the protrusions (Fig. 2D). The protrusions stained strongly with the fluorescent cationic dye acridine orange, which accumulates within the apoptotic cell nucleus and cytoplasm but not in necrotic or viable cells (Fig. 2E) (35). Additional experiments with the fluorescently labeled transgenic zebrafish lines Tg(*krt8*:GFP) (36) and Tg(*fli1a*:EGFP) (37) showed that the F11-induced protrusions were composed largely of epithelial cells (Fig. 2F) and devoid of leukocytes and endothelial cells (Fig. 2G). These epithelial protrusions presumably arise in response to an as-yet-undefined toxin or another factor(s) that is expressed by F11 but missing from CFT073.

### Ciprofloxacin treatment is only effective for a short window of time that varies, depending on the infecting strain.

**FIG 3:**
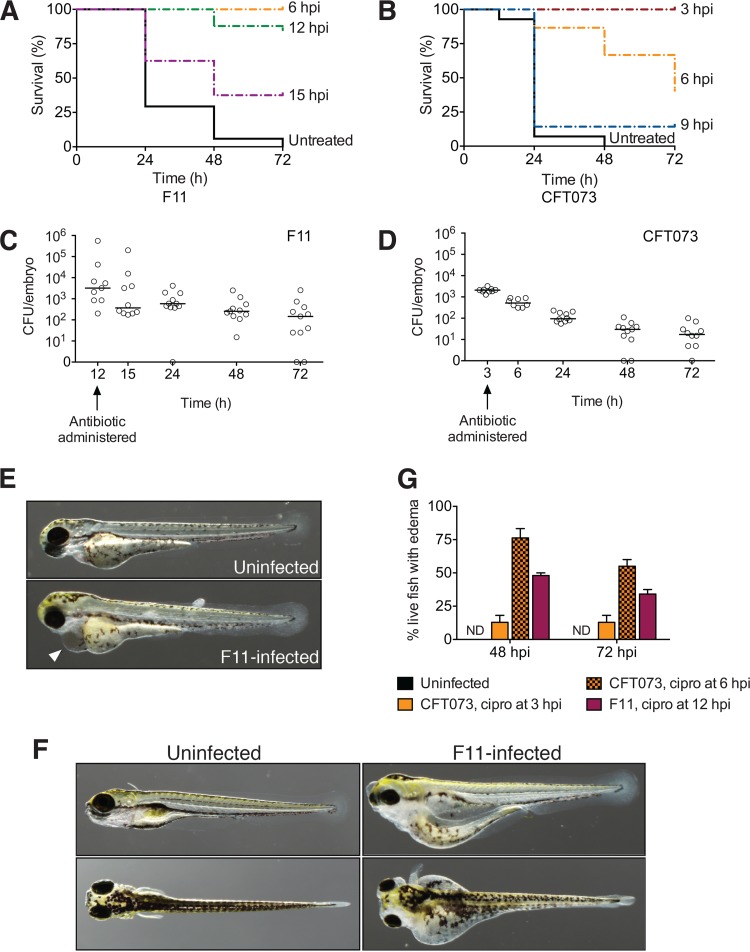
Survival of ExPEC-infected embryos varies with ciprofloxacin treatment. (A and B) Kaplan-Meier survival curves of zebrafish embryos injected via the circulation valley with F11 (A) or CFT073 (B) and then treated with ciprofloxacin at the times indicated; *n* = 15 to 20 embryos. Black solid lines show untreated controls. (C and D) Bacterial titers recovered from F11 (C)- and CFT073 (D)-infected embryos before and after treatment with ciprofloxacin at 12 or 3 hpi, as indicated. Bars denote the median values of the groups; *n* ≥9 embryos pooled from two independent experiments. (E and F) Uninfected controls and F11-infected embryos at 24 (E) and 72 (F) hpi. Both control and F11-infected zebrafish were treated with ciprofloxacin beginning at 12 hpi. The arrowhead in panel E indicates the presence of pericardial edema in a surviving F11-infected embryo. (G) Percentages of surviving embryos with overt signs of edema at 48 and 72 hpi of the bloodstream with F11 or CFT073. Ciprofloxacin was administered at 3, 6, or 12 hpi, as indicated. Uninfected controls were treated with ciprofloxacin at the same time points. Bars represent mean data ± the standard error of the mean from two independent experiments; total *n* = 30 to 40 fish. ND, not detected.

The survival of sepsis patients is often contingent upon rapid diagnosis and the timely delivery of appropriate antibiotics—for every hour that antibiotic treatment is delayed, there is a 6 to 7% increased chance of patient death (38). Even with adequate antimicrobial therapies that curtail bacterial growth, complications such as edema, immunosuppression, and coagulation defects are frequent (39–41). To determine how systemically infected zebrafish respond to antibiotic treatment, we administered ciprofloxacin at various time points postinoculation with CFT073 or F11. Ciprofloxacin was chosen because it is of use clinically for the treatment of Gram-negative sepsis, and particularly for sepsis arising from the gastrointestinal or urinary tract (42, 43). Additionally, ciprofloxacin readily permeates the cell, making it easy to administer by placing infected zebrafish embryos in water containing the antibiotic. When F11-infected embryos were treated with 50 µg/ml ciprofloxacin at 6 hpi, the antibiotic was able to rescue all of the embryos from lethal infection (Fig. 3A). When given at 12 hpi, ciprofloxacin was only slightly less effective, but a delay of just another 3 h resulted in greatly increased mortality rates.

In sharp contrast to results obtained with the F11-infected embryos, the complete rescue of all CFT073-infected zebrafish required that we administer ciprofloxacin at a much earlier time point (3 hpi, Fig. 3B). The addition of ciprofloxacin to CFT073-infected fish at 6 hpi decreased the survival rate to less than 30%, while waiting just another 3 h made it almost entirely ineffective (Fig. 3B). Of note, CFT073 and F11 are similarly susceptible to ciprofloxacin, as measured by disc diffusion antibiotic sensitivity assays (see Fig. S2 in the supplemental material). Furthermore, within zebrafish embryos, the growth of both pathogens is effectively inhibited by addition of the antibiotic (Fig. 3C and D). Taken together, these results demonstrate that, as with human sepsis patients, the early administration of antibiotic therapy is crucial for the survival of infected zebrafish and that even small delays in treatment can drastically increase mortality rates. In addition, these data show that ExPEC isolates that are similarly sensitive to an antibiotic can have drastically different effects on disease outcome when exposed to the same antibiotic within the host environment.

10.1128/mSphere.00062-16.3 Figure S2  CFT073 and F11 are similarly sensitive to ciprofloxacin *in vitro*. (A and B) Representative images of ciprofloxacin disc diffusion assays on LB plates incubated overnight at 28.5°C (A) or 37°C (B). Each disc contains 5 µg of ciprofloxacin. Download Figure S2, PDF file, 0.6 MB. Copyright © 2016 Barber et al. 2016 Barber et al. This content is distributed under the terms of the Creative Commons Attribution 4.0 International license.

### Infected embryos often develop profound edema following rescue with ciprofloxacin.

In our studies with ciprofloxacin given to F11-infected zebrafish at 12 hpi, we noticed that the surviving animals often displayed marked pericardial edema at 24 hpi (Fig. 3E). By 48 hpi, approximately 50% of the surviving embryos developed profound widespread progressive edema (Fig. 3F and G). These embryos were not retained beyond 96 hpi, but it is unlikely that they would have successfully survived to adulthood. We also observed similarly severe edema in many of the surviving CFT073-infected fish that were dosed with ciprofloxacin at 3 or 6 hpi, while no edema was observed in uninfected embryos treated with ciprofloxacin (Fig. 3G). These results indicate that complications like subcutaneous and body cavity edema, which is not uncommon in human sepsis patients (39, 40, 44), can also develop in infected zebrafish despite effective inhibition of pathogen growth.

### Lethal ExPEC isolates can trigger divergent host transcriptional responses.

**FIG 4:**
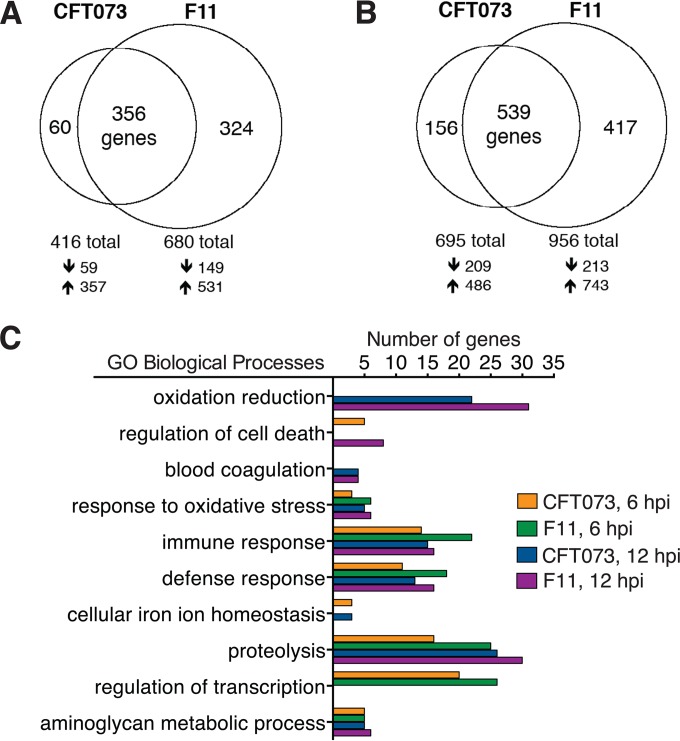
Equally lethal ExPEC isolates trigger distinct host responses. (A and B) Venn diagrams indicating the numbers of host genes that are differentially expressed in zebrafish embryos at 6 (A) or 12 (B) hpi with CFT073 versus F11, as determined by microarray analysis. The number of differentially expressed transcripts was calculated for each data set by gating on probes with ≥2-fold changes relative to mock-infected (PBS-injected) controls and *P* ≤ 0.05. Results from multiple probes that mapped to the same gene were combined to generate nonredundant lists of the differentially expressed genes. Arrows below the Venn diagrams denote transcripts that were up- or downregulated relative to controls. (C) The numbers of differentially expressed host genes in a selected list of enriched GO terms, as determined by DAVID.

The differential pathophysiological effects that F11 and CFT073 have on the zebrafish host were examined more closely by transcriptional profiling. With a previously described Agilent microarray enriched with probes specific for zebrafish homologues of mammalian immunity genes (45–47), the host response to each ExPEC strain was surveyed at 6 and 12 hpi. The 6-h time point represents the more acute phase of infection, while the 12-h time point reflects a later stage, where the two strains have established a solid foothold and replicated to high titers within the zebrafish host (see Fig. 1B). For these experiments, groups of 18 to 20 embryos were injected with CFT073, F11, or a similar volume of sterile PBS as a control. RNA was then isolated from each pool of embryos at 6 and 12 hpi and processed for analysis with two-color 44k microarrays. Each experiment was repeated in biological quadruplicate. Gating on probes that were differentially expressed at least 2-fold up or down compared to PBS-injected fish and using a *P* value of ≤0.05, we found that infection with F11 altered mRNA levels for nearly a third more genes than CFT073 at both 6 and 12 hpi (Fig. 4A and B; see also Data Set S1 in the supplemental material). While infection with both strains changed the expression of a large number of overlapping host genes, F11 infection also induced an almost equal-sized cohort of additional host genes that was not seen in CFT073-infected fish. Twenty-two transcripts were identified that were repressed in CFT073-infected embryos but induced in fish infected with F11 (see Data Set S1). No transcripts with the opposite pattern of expression were observed.

10.1128/mSphere.00062-16.1 Data Set S1  Excel file listing transcripts that are differentially expressed by zebrafish embryos during infection with CFT073 and/or F11 at 6 and 12 hpi. The gene transcripts listed show ≥2-fold changes relative to PBS-injected controls (*P* ≤ 0.05). Results obtained with all of the relevant probes available on the chip for the genes specified are shown. The “Opposite Expression” tab lists transcripts that show opposite directions of induction in CFT073- versus F11-infected embryos at either 6 or 12 hpi. Download Data Set S1, XLSX file, 0.7 MB. Copyright © 2016 Barber et al. 2016 Barber et al. This content is distributed under the terms of the Creative Commons Attribution 4.0 International license.

The biological attributes of the host genes that are differentially expressed in response to F11 and CFT073 at 6 and 12 hpi were assessed by gene ontology (GO) and KEGG pathway enrichment analysis with DAVID bioinformatics resources (48). GO categories that were enriched at both time points with both ExPEC isolates included those dealing with host response to oxidative stress (e.g., peroxidases), immune and defense responses (e.g., cytokine expression and complement components), proteolysis (e.g., caspases and matrix metalloproteinases), and aminoglycan metabolism (e.g., peptidoglycan recognition protein 5) (Fig. 4C). In contrast, the number of genes within the GO category for regulators of transcription was significantly elevated only at 6 hpi, whereas GO categories linked with oxidation reduction (redox) reactions and blood coagulation were not enriched until 12 hpi. The latter group includes upregulated genes that encode coagulation factor IIIb (*f3b*) and the α, β, and γ chains of fibrinogen. These gene products are functionally well conserved among vertebrates as mediators of thrombosis and have been implicated in DIC during sepsis (41, 49–51).

In many cases, the number of differentially expressed genes in each enriched GO category was greater for the F11-infected fish (Fig. 4C). This trend was also observed by KEGG pathway analysis (see Fig. S3 in the supplemental material). Common pathways affected by F11 and CFT073 at both 6 and 12 hpi include those involved in apoptosis; proteasome activities; arachidonic acid metabolism; and TLR, mitogen-activated protein (MAP) kinase, and cytokine signaling. At this time point, F11 also caused significant transcriptional changes in several genes that encode proteins within the p53 pathway (e.g., cyclin B1, GADD45 homologues, and Sestrin-2), which has a central role as a regulator of host cell cycle arrest and apoptosis in response to stress (52).

10.1128/mSphere.00062-16.4 Figure S3  CFT073 and F11 have differential effects on host biological pathways. Quantification of differentially expressed genes (change, ≥2-fold; *P* ≤ 0.05) within all KEGG pathways that are significantly affected in response to systemic infection of zebrafish embryos with CFT073 and/or F11 as determined by DAVID. Download Figure S3, PDF file, 0.1 MB. Copyright © 2016 Barber et al. 2016 Barber et al. This content is distributed under the terms of the Creative Commons Attribution 4.0 International license.

### Zebrafish embryos infected with lethal ExPEC isolates have transcriptional responses and pathologies like those seen in human sepsis.

In humans with sepsis or related syndromes like endotoxemia, there is often a large amount of variation in the gene expression data sets obtained from different studies (53). However, general trends in these data sets are discernible and are reiterated, in large part, in our zebrafish infection model (Table 1). These include the upregulation of pattern recognition receptors like TLR5, as well as components of the NF-κB, MAP kinase, and Jak-STAT signaling pathways. Activation of these pathways can stimulate the expression of multiple cytokines, chemokines, and other immunomodulatory factors. The high-level expression of numerous cytokines and other inflammatory mediators induced in zebrafish by both F11 and CFT073 is reminiscent of the cytokine storms seen in human patients during sepsis. Major inflammatory cytokines that are substantially elevated both in our assays and in human patients with sepsis-like syndromes include tumor necrosis factor alpha (TNF-α), TNF-β, interleukin-1β (IL-1β), IL-6, IL-8, IL-12, and IL-17 (49, 54–59). During human sepsis, as in many inflammatory diseases, there is an imbalance between pro- and anti-inflammatory mediators (59). We observe a similar situation in our zebrafish model, as evidenced by the simultaneous upregulation of multiple proinflammatory signals along with several key anti-inflammatory factors, including IL-10, SOCS1 and -3, IRAK3, and galectin-1 (Table 1). Zebrafish infected with lethal ExPEC isolates also display pathologies seen in human sepsis, such as endothelial leakage and tachycardia (for further details, see Fig. S4 in the supplemental material). In total, these observations demonstrate that ExPEC-infected zebrafish have altered gene expression patterns and develop overt pathologies on a par with those that are often observed in humans with sepsis and related syndromes.

10.1128/mSphere.00062-16.5 Figure S4  ExPEC induces vascular leakage and tachycardia in zebrafish embryos. (A) Endothelial leakage in infected and control PBS-injected embryos at 9 hpi. Dextran leakage was quantified by calculating the ratio of the fluorescence intensity within the myotomes to that in the underlying vasculature. Data are the mean ± the standard error of the mean of two independent experiments; *n* = 9 to 15 embryos with three measurement sites used per fish. (B) Heart rates (beats per minute) of infected and PBS-injected control zebrafish embryos at 6 hpi. Each bar represents the mean ± the standard error of the mean of two independent experiments; *n* = 10 to 12 embryos. *P* values were determined by unpaired Student *t* test. Also shown is additional information related to the image shown. Zebrafish embryos infected with lethal ExPEC isolates have pathologies like those seen in human sepsis. Download Figure S4, PDF file, 0.3 MB. Copyright © 2016 Barber et al. 2016 Barber et al. This content is distributed under the terms of the Creative Commons Attribution 4.0 International license.

**TABLE 1:** Gene sets that are differentially expressed in zebrafish embryos because of systemic infection with ExPEC are functionally similar to many of those that are changed in human patients with sepsis

Functional category	Gene products^a^	Links to human sepsis^b^
Signal transduction		55–58, 101
Pattern recognition receptors	TLR5, MARCO scavenger receptor, MyD88, IRAK4, IRF7, TRAF1/2b/3	
NF-κB	Rel, NF-κB2, NF-κBIa (IκBα), Bcl3, TRAF1/2b, RIPK2	
MAP kinase	Fos, Jun, ATF-3, MEKK5, TRAF1/2b, GADD45A/B	
Jak-STAT	Jak1, STAT4, STAT1b, STAT3, IRF9	
Proinflammatory mediators	TNF-α/β, TNF receptors (TNFRSF1a and TNFRSF9a), IL-1β, IL-12, IL-17, IL-8	49, 55, 56, 58
Anti-inflammatory factors	SOCS1/3, IL-10, galectin-1, IRAK3 (IRAK-M)	55–58, 101, 102
Acute-phase proteins	SAA1, HAMP1, haptoglobin, hemopexin	49, 56, 57, 66, 68, 101, 103
Coagulation and complement	Coagulation factor IIIb, fibrinogen, complement factor B, clusterin, complement components C3b, C3c, C4-2, C6, C7-1	56, 57, 101
Protease activities	MMP9/13/30, proteasome subunits, cathepsin C/H, carboxypeptidase A4, ADAM8a, SERPINB1/5	56, 57, 67
ROS^c^ generation and management	Neutrophil cytosolic factor 1 (p47-PHOX), cytochrome *b*-245 (p22-PHOX), glutathione peroxidase 1b, thioredoxin, UCP2, GADD45A/B, metallothionein 2, solute carrier family 30/39	50, 55, 56, 58, 104
Apoptosis	Caspase 8, Fas, MEKK5 (ASK-1), CFLAR, TRAF1/2b, galectin-1, RIPK2	56, 55, 57, 58
Arachidonic acid metabolism	Prostaglandin-endoperoxide synthase 1 (COX-1), prostaglandin-endoperoxide synthase 2a/b (COX-2), glutathione peroxidase 1b, epoxide hydrolase 2, cytochrome p450s, cholesterol 25-hydroxylase	56, 105, 106

a Transcript levels for the gene products indicated are significantly increased ≥2-fold (*P* < 0.05) in F11- and/or CFT073-infected zebrafish embryos at 6 and/or 12 hpi, relative to those in mock-infected controls.

b The reference numbers listed are those of studies that implicate the specified functional categories or selected gene products in human sepsis and related syndromes but do not necessarily include all of the pertinent publications.

c ROS, reactive oxygen species.

### F11 and CFT073 have differential effects on TLR signaling and associated inflammatory responses.

Our KEGG analysis indicated that F11 and CFT073 have especially sizeable effects on TLR signaling pathways (see Fig. S3 in the supplemental material). For a map that illustrates how F11 and CFT073 influence the transcription of specific TLR genes, associated signaling factors, and various downstream targets, see Fig. S5 in the supplemental material. Both pathogens alter the expression of several key components within canonical MyD88-dependent and MyD88-independent TLR signaling cascades, as well as intersecting and overlapping MAP kinase, Jak-STAT, interferon (IFN), and TNF signaling pathways. Although F11 and CFT073 have similar effects on the expression of many of the inflammatory mediators depicted in Fig. S5 in the supplemental material, our array data indicate that the pathogens can also elicit conspicuously divergent inflammatory responses. Examples of this phenomenon are seen with two conserved cytokines—the macrophage-activating cytokine IFN-γ2 (*ifng1-2*), which is induced by F11 but repressed by CFT073, and the anti-inflammatory factor IL-10, which is markedly upregulated by CFT073 but less so by F11.

10.1128/mSphere.00062-16.6 Figure S5  CFT073 and F11 have overlapping but distinct effects on the expression of TLR pathway genes and downstream inflammatory-mediator-encoding genes. The diagram shows differentially expressed host genes at 6 and 12 hpi with CFT073 or F11. Upregulated genes are yellow, while those that are downregulated are purple (change up or down, ≥2.0-fold; *P* ≤ 0.05). Download Figure S5, PDF file, 0.5 MB. Copyright © 2016 Barber et al. 2016 Barber et al. This content is distributed under the terms of the Creative Commons Attribution 4.0 International license.

**FIG 5:**
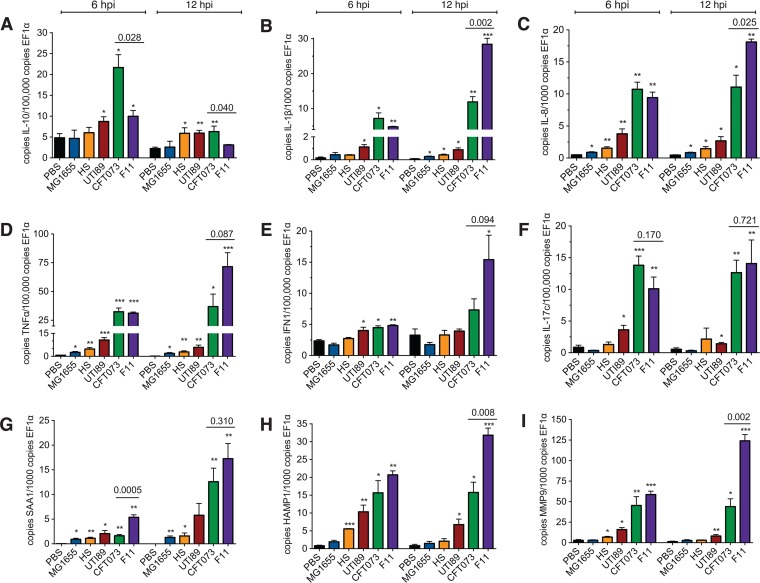
Variable expression of cytokines and host defense genes in response to lethal and nonlethal *E. coli* strains. (A to I) qRT-PCR analysis of the specified cytokine transcripts at 6 and 12 hpi with PBS or ~1,000 CFU of the *E. coli* strains indicated. Each bar represents mean results ± the standard error of the mean from three or four pools of ≥16 embryos. A qRT-PCR assay of each pool was carried out with technical duplicates. *, *P* ≤ 0.05; **, *P* ≤ 0.01; ***, *P* ≤ 0.001 (versus PBS-injected controls, as determined by Student’s *t* test). *P* values for F11- versus CFT073-infected samples are also indicated.

**FIG 6:**
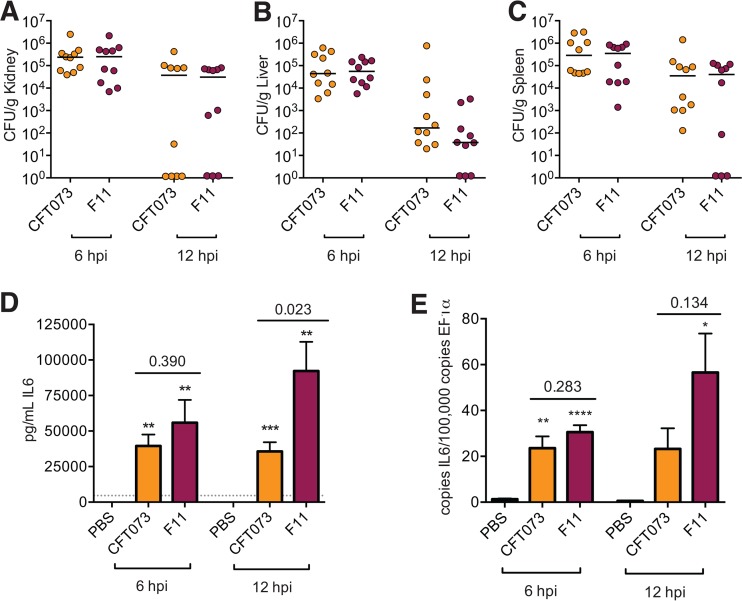
The differential effects of CFT073 and F11 on host responses are recapitulated in a mouse model of sepsis. (A to C) Bacterial titers recovered from the kidneys, livers, and spleens of outbred Swiss-Webster mice at 6 and 12 hpi with ~10^8^ CFU of CFT073 or F11 via subcutaneous injection. Data from two independent experiments were pooled; total *n* = 10 mice per time point. Horizontal lines indicate median values. CFT073 and F11 titers were not significantly different at any time point, as determined by the Mann-Whitney U test. (D) Serum IL-6 levels in mice at 6 and 12 hpi with PBS, CFT073, or F11, as determined by enzyme-linked immunosorbent assay. Data are representative of mean results ± the standard error of the mean from two independent experiments; *n* = 5 mice. The gray dotted line shows the limit of quantification. *, *P* ≤ 0.05; **, *P* ≤ 0.01; ***, *P* ≤ 0.001 (versus PBS-injected controls, as determined by Student’s *t* test). *P* values for F11- versus CFT073-infected mice are also indicated. (E) IL-6 transcript levels in zebrafish embryos at 6 and 12 hpi with PBS, F11, or CFT073, as determined by qRT-PCR. Each bar represents the mean value ± the standard error of the mean of three independent experiments, each with 15 to 20 embryos. A qRT-PCR assay of each pool was performed with technical duplicates.

Quantitative reverse transcription (qRT)-PCR was used to validate a subset of our microarray results and to explore the host transcriptional responses to additional *E. coli* strains (Fig. 5). In agreement with our microarray data, qRT-PCR indicated that CFT073 induced significantly higher levels of IL-10 than did F11 at both 6 and 12 hpi (Fig. 5A). Following a spike at 6 hpi, IL-10 transcript levels in CFT073-infected fish were reduced by 12 hpi to levels seen in embryos injected with either the gut isolate HS or nonlethal ExPEC strain UTI89. Interestingly, the IL-10 mRNA levels induced by HS and UTI89 at 12 hpi were still significantly greater than those observed in F11-infected fish and in control animals inoculated with either PBS or K-12 strain MG1655. The situation was quite different when transcript levels for the proinflammatory cytokines IL-1β and IL-8 were quantified. Relative to the nonlethal strains, CFT073 and F11 enhanced the expression of IL-1β and IL-8 similarly at 6 hpi, but by 12 hpi, the transcript levels for these cytokines in F11-infected embryos were markedly higher than those in fish infected with CFT073 (Fig. 5B and C). Similar trends in the proinflammatory cytokines TNF-α, IFN-1, and IL-6 in F11- and CFT073-infected fish were observed (Fig. 5D and E and 6E). In contrast, F11 and CFT073 had comparable effects on the expression of another proinflammatory cytokine, IL-17c, which was strikingly elevated at both 6 and 12 hpi in response to the two lethal pathogens (Fig. 5F).

The differing effects of F11 and CFT073 on cytokines like IL-1β and IL-8 were in part recapitulated when we used qRT-PCR to survey the expression levels of three other host defense genes—*saa1*, *hamp1*, and *mmp9*. Transcription of the gene encoding serum amyloid A1 (SAA1), which is an acute-phase protein with antimicrobial activity (60–63), was significantly induced by all of the *E. coli* strains tested, relative to that in PBS-injected controls (Fig. 5G). However, F11 caused a more rapid and higher level of induction than any of the other strains, including CFT073. Transcript levels for hepcidin (HAMP1), which is an antibacterial peptide that also functions as a key regulator of iron homeostasis (64), were significantly elevated at 6 hpi in response to all of the strains except MG1655 (Fig. 5H). By 12 hpi, *hamp1* expression was notably higher in the F11-infected embryos than in all other samples, including the CFT073-infected fish. A comparable expression pattern was seen with the matrix metalloproteinase MMP9, a secreted protease that facilitates the migration of leukocytes to sites of infection (Fig. 5I) (65). Of note, serum SAA1, HAMP1, and MMP9 levels are elevated during human sepsis and have been investigated as prognostic biomarkers (Table 1) (66–68).

Overall, these qRT-PCR results are in close agreement with our microarray data, demonstrating that similarly lethal ExPEC isolates like F11 and CFT073 can elicit distinct, though overlapping, inflammatory responses during systemic infections. This phenomenon was mirrored in a mouse model of sepsis in which we focused on IL-6 expression. IL-6 is a marker of sepsis severity, with elevated levels of this cytokine during early sepsis correlating with increased mortality rates in both humans and mice (69–71). Following subcutaneous inoculation of adult outbred Swiss-Webster mice with 10^8^ CFU of either CFT073 or F11, both pathogens disseminate systemically and kill 90 to 100% of the host animals within 24 h. At 6 and 12 hpi, we detected no significant differences in the titers of the two ExPEC isolates within the kidneys, liver, or spleen (Fig. 6A to C). Relative to PBS-injected controls, both pathogens induce the production of IL-6, but by 12 hpi, the levels of IL-6 in the sera of F11-infected mice were significantly higher than those in CFT073-infected animals (Fig. 6D). A similar, though less distinct, pattern of IL-6 expression was also seen in F11- and CFT073-infected zebrafish embryos (Fig. 6E), paralleling our findings on other proinflammatory cytokines.

### Flagellins from CFT073 and F11 differentially modulate inflammatory responses during sepsis.

**FIG 7:**
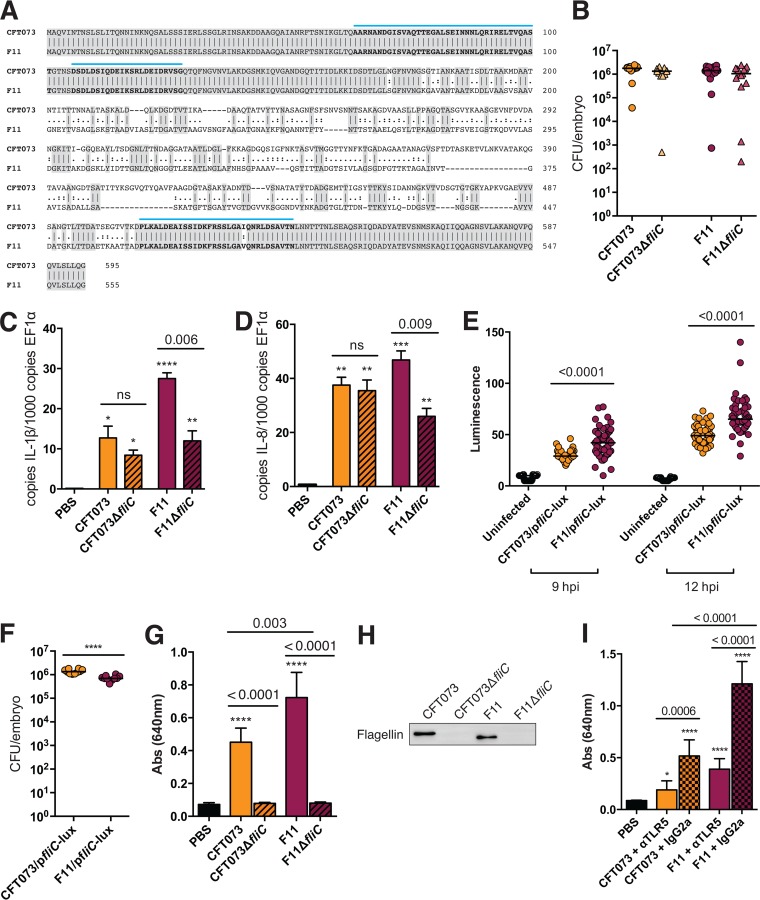
Flagellins from CFT073 and F11 have differential effects on TLR5 activation. (A) Alignment of the sequences of the FliC proteins of CFT073 (H1) and F11 (H31). The sequences are 67.3% similar. Identical residues are shaded gray. Predicted TLR5 binding regions are indicated by blue lines and bold text. (B) Bacterial burdens in infected embryos at 12 hpi with ~1,000 CFU of the strains indicated. Data were pooled from two independent experiments. Bars denote median values; *n* = 10 embryos. No significant differences between wild-type and *fliC* mutant strains were detected by Mann-Whitney U tests. (C, D) qRT-PCR analysis of the cytokine transcripts specified at 12 hpi with PBS or ~1,000 CFU of the *E. coli* strains indicated. Bars indicate mean results ± the standard error of the mean from three pools of 18 to 20 embryos. qRT-PCR for each pool was carried out with technical duplicates. *, *P* ≤ 0.05; **, *P* ≤ 0.01; ***, *P* ≤ 0.001 (versus PBS-injected controls, as determined by Student’s *t* test). *P* values for wild-type versus *fliC* mutant samples are also indicated. (E) Levels of FliC expression in infected embryos at 9 and 12 hpi, as determined with the p*fliC*-lux reporter construct. Lines mark median values; *n* = 30 to 50 embryos pooled from two independent experiments. *P* values were calculated by Mann-Whitney U tests. (F) Bacterial burdens in infected embryos at 12 hpi with ~2,000 to 2,500 CFU of CFT073 or F11 carrying p*fliC*-lux. Larger inoculation doses were used to compensate for the partial loss of fitness of strains carrying p*fliC*-lux. Data from two independent experiments were pooled. Lines mark median values; *n* = 10 embryos. ****, *P* ≤ 0.0001 (as calculated by Mann-Whitney U test). (G) Immunoblot assay showing adjusted levels of FliC in flagellar preparations from CFT073, CFT073Δ*fliC*, F11, and F11Δ*fliC*. (H) TLR5 stimulation by flagellar preparations, as measured with HEK-Blue mTLR5 reporter cells. Bars represent mean results ± 95% confidence intervals from two independent experiments with three replicates. ****, *P* ≤ 0.0001 (versus the PBS control, as determined by Student’s *t* test). *P* values for wild-type versus *fliC* mutant samples and CFT073 versus F11 are also indicated. (I) TLR5 stimulation by semipurified flagella in the presence of a TLR5 neutralizing or control antibody, as measured with HEK-Blue mTLR5 reporter cells. Bars represent mean results ± 95% confidence intervals from three independent experiments with three replicates. *, *P* ≤ 0.05; **, *P* ≤ 0.01; ***, *P* ≤ 0.001; ****, *P* ≤ 0.0001 (versus the PBS control, as determined by Student’s *t* test). *P* values for the TLR5 neutralizing antibody versus the control antibody and CFT073 versus F11 are also indicated.

CFT073 and F11 share the same type 2 (K2) capsule and O6 lipopolysaccharide (LPS) antigens but possess distinct flagellar serotypes (H1 versus H31, respectively). The flagellin (FliC) proteins from CFT073 and F11 are only 67.3% similar at the protein coding level, with each being composed of well-conserved N- and C-terminal domains separated by a variable middle domain that is responsible for generating serotype diversity (Fig. 7A). This domain architecture is common among bacterial flagellin proteins and provides the basis for flagellin recognition by TLR5 (72–74). Regions within the conserved termini of FliC that are predicted to be bound by TLR5 are nearly identical in the CFT073 and F11 sequences, with the exception of a single conservative isoleucine-to-valine change. Considering the stimulatory effects of both CFT073 and F11 on the transcription of TLR5 and associated signaling factors (see Fig. S5 in the supplemental material), we wished to determine the contributions of the FliC variants to the infection process and host responses. Deletion of *fliC* from CFT073 and F11 rendered both pathogens immobile on swim agar plates (see Fig. S6 in the supplemental material) but had no obvious effects on the lethality of the strains or on bacterial titers recovered from the zebrafish host at 12 hpi (Fig. 7B). At this time point, CFT073Δ*fliC* induced levels of the proinflammatory cytokines IL-1β and IL-8 similar to those induced by its wild-type counterpart, whereas F11Δ*fliC* was markedly less inflammatory than the wild-type F11 strain (Fig. 7C and D).

10.1128/mSphere.00062-16.7 Figure S6  Functional verification and complementation of *fliC* mutants. (A and B) Deletion of *flic* from F11 (A) and CFT073 (B) renders both pathogens immobile on 0.1% LB agar plates. Wild-type strains are also pictured as controls. (C) Complementation of F11Δ*fliC* with pBF14 restores motility on 0.1% LB agar plates, while the mutant carrying the empty vector pGEN remains immobile. (D) Complementation of CFT073Δ*fliC* with pBF15 restores motility on 0.1% LB agar plates, while the mutant carrying the empty vector pGEN remains immobile. Download Figure S6, PDF file, 0.1 MB. Copyright © 2016 Barber et al. 2016 Barber et al. This content is distributed under the terms of the Creative Commons Attribution 4.0 International license.

The levels of flagellin expression by CFT073 and F11 during infection of the zebrafish host were examined with a low-copy reporter construct in which the *luxCDABE* gene cluster encoding bacterial luciferase is transcriptionally fused with the conserved *fliC* promoter (P*_fliC_*-*lux*) (75). Significantly higher levels of *fliC* expression were detected in zebrafish infected with F11/p*fliC*-lux than in those infected with CFT073/p*fliC*-lux (Fig. 7E). Titers of the recombinant strains present at 12 hpi were comparable, though marginally fewer F11/p*fliC*-lux bacteria were recovered (Fig. 7F). With a TLR5 reporter cell line (HEK-Blue mTLR5 cells), we found that semipurified preparations of flagella from F11 were significantly more stimulatory than preparations from CFT073 (Fig. 7G), despite the presence of similar amounts of FliC (as verified by immunoblotting, Fig. 7H). In these assays, HEK-Blue mTLR5 cells treated with preparations from CFT073Δ*fliC* or F11Δ*fliC* showed no significant activation above the baseline (PBS-treated cells). Further confirming the specificity of the measured responses, we found that the addition of an anti-TLR5 neutralizing antibody, but not an isotype control, significantly inhibited stimulation of the TLR5 reporter cells by flagella from both CFT073 and F11 (Fig. 7I). In total, these data suggest that the divergent inflammatory responses seen in F11- and CFT073-infected zebrafish are in part attributable to differences in the levels of FliC expression by these two pathogens, as well as contrasting stimulatory effects of the two FliC variants on TLR5 signaling.

## DISCUSSION

Sepsis patients often present with wide-ranging disease symptoms and are notoriously difficult to manage because they do not respond homogeneously to treatment (53). In septic individuals, the infecting microbes are commonly viewed as generic inducers of inflammation while the host background is considered the primary variable affecting disease progression and outcome. Here, with a model system in which bacteria are injected into the zebrafish embryo bloodstream, we found that only a limited subset of ExPEC isolates is able to persist within the host and cause overt sepsis-like disease. In addition, our results show that similarly lethal bacterial isolates belonging to the same species can affect the development of sepsis in markedly different ways, irrespective of host background characteristics.

The diversity of host responses elicited by F11, CFT073, and other ExPEC isolates in our zebrafish model reflects many of those seen in human patients with bacteremia or sepsis. Overt pathophysiological changes observed within both infected zebrafish and human sepsis patients include tachycardia, vascular leakage, edema, and signs of leukopenia. In addition, though we did not assess clotting abnormalities directly, our microarray results show that the ExPEC strains do have significant effects on host coagulation factors at 12 hpi, suggesting that the pathogens may cause dysregulation of clotting cascades that could lead to problems like DIC within zebrafish.

In our assays, F11 altered the transcription of nearly a third more host genes than CFT073 at 6 and 12 hpi, while simultaneously inducing higher-level expression of many proinflammatory cytokines and other immunomodulatory factors. This trend was also observed in serum IL-6 levels in a mouse sepsis model. In comparison with CFT073-infected zebrafish, those animals infected with F11 often appeared qualitatively more ill and frequently developed apoptotic epidermal protrusions (see Fig. 2C to G). These protrusions, which resemble lesions seen on zebrafish embryos infected systemically with *Enterococcus faecalis* (76), may be manifestations of the cutaneous lesions seen in some human patients with bacteremia or sepsis (77, 78). The factors that promote their formation are not yet defined. Overall, CFT073 was less inflammatory than F11 and is arguably a stealthier pathogen, stimulating the expression of more anti-inflammatory factors like IL-10 and producing fewer overt signs of illness during the initial stages of infection. From these results, we posit that the ability of different ExPEC isolates to elicit dissimilar host inflammatory responses in human patients contributes to the heterogeneity of symptoms experienced by septic individuals.

Disparity in the observed host responses to F11 and CFT073 correlates with variations in the levels of flagellin expression by these two ExPEC isolates and divergent stimulatory effects of the FliC variants on TLR5 signaling (see Fig. 7). The involvement of flagellin as a regulator of host responses during sepsis has precedents. Flagellin can be detected in the plasma of sepsis patients at concentrations ranging from 2 to 20 ng/ml, with higher levels being associated with longer durations of septic shock (79). In mouse burn wound models of sepsis, the administration of flagellin can enhance the antibacterial activities of neutrophils and the delivery of antiflagellin antibodies can promote host survival (80, 81). In both our zebrafish model and human patients with sepsis-like syndromes, the flagellin receptor TLR5 is among the most highly induced pattern recognition receptors (56, 58, 82). Allelic variants of TLR5 may predispose infants to sepsis, and high-level expression of TLR5 in septic individuals is positively linked with more severe disease (82–84).

The capacity of distinct flagellar serotypes to differentially activate TLR5, as demonstrated in this study, indicates an unanticipated mechanism by which different ExPEC isolates may elicit various inflammatory responses in septic individuals and during other infections. The predicted canonical TLR5-binding sites within the FliC variants from CFT073 and F11 are nearly identical, save for one residue (I528 in CFT073 or V488 in F11) that sits on the backside of the C-terminal α-helix that interfaces with TLR5 (73, 74). This change, as well as multiple synonymous single-nucleotide polymorphisms present within coding sequences for the conserved termini of the FliC variants, could potentially affect signaling via TLR5. Though the hypervariable middle domain of FliC does not make direct contact with TLR5, previous studies indicate that this domain may also modulate cytokine responses (85, 86). The ability of different versions of FliC to elicit contrasting host responses suggests that some bacteria may utilize flagella as immunomodulators, expanding their function beyond motility. These findings also suggest that specific FliC variants could be engineered as regulators of host inflammatory responses with possible therapeutic value.

Since 2000, the rates of hospitalization for sepsis have doubled and associated costs have skyrocketed in the United States to greater than $20 billion annually (87). In the clinic, effective treatment of sepsis typically requires early diagnosis and timely delivery of fluids to maintain blood pressure plus broad-spectrum antibiotics to control the infecting bacteria (38, 88). Results presented here reiterate the importance of timely antibiotic delivery for the survival of a septic host (Fig. 3). Furthermore, our data show that the window during which antibiotic therapy can effectively rescue the host can vary greatly, even when ExPEC strains that are similarly sensitive to the drug are compared. Adding to the complexity of factors that can confound the assessment and treatment of sepsis patients is the ongoing rise of multidrug-resistant (MDR) pathogens, including MDR ExPEC strains (89, 90).

Recently, the suitability of animal models to address the complex pathophysiological mechanisms that underlie sepsis in humans has been debated (91–93). We believe that the use of outbred zebrafish, as employed here, can be a valuable tool for understanding the vagaries of sepsis and related pathologies. Zebrafish embryos are amenable to fairly high-throughput pharmacological and genetic screens, and work by our group and others has already demonstrated the utility of zebrafish as a tool for identifying and functionally defining bacterial and host factors that are of importance during infection in the bloodstream (14, 15, 17, 29, 94–96). One potential caveat to using zebrafish as a model of sepsis is that they are considerably less responsive to LPS than humans are (97–99). However, considering the tractability of the zebrafish infection model and its ability to recapitulate important aspects of human disease, as shown here, we expect that it will provide an excellent platform for the discovery and assessment of improved therapies for sepsis and associated sequelae.

## MATERIALS AND METHODS

For a full description of the strains, plasmids, and Materials and Methods used in this study, see Tables S1 and S2 and Text S1 in the supplemental material. The animals used in this study were handled in accordance with University of Utah- and IACUC-approved protocols (protocol no. 10-02014) following standard guidelines described at www.zfin.org and in the Guide for the Care and Use of Laboratory Animals, 8th Edition.

10.1128/mSphere.00062-16.8 Table S1  Bacterial strains and plasmids used in this study. Download Table S1, PDF file, 0.04 MB. Copyright © 2016 Barber et al. 2016 Barber et al. This content is distributed under the terms of the Creative Commons Attribution 4.0 International license.

10.1128/mSphere.00062-16.9 Table S2  Primers used in this study. Download Table S2, PDF file, 0.04 MB. Copyright © 2016 Barber et al. 2016 Barber et al. This content is distributed under the terms of the Creative Commons Attribution 4.0 International license.

10.1128/mSphere.00062-16.10 Text S1  Supplemental materials and methods and references for supplemental files. Download Text S1, PDF file, 0.2 MB. Copyright © 2016 Barber et al. 2016 Barber et al. This content is distributed under the terms of the Creative Commons Attribution 4.0 International license.

### Infection of zebrafish embryos.

At 48 hpf, embryos were manually dechorionated, briefly anesthetized with 0.77 mM ethyl 3-aminobenzoate methanesulfonate salt (tricaine; Sigma-Aldrich), and embedded in 0.8% low-melting-point agarose (Mo Bio Laboratories) without tricaine. After the agarose solidified, embryos were immersed in E3 medium lacking methylene blue. Prior to injection, 1 ml of bacterial culture was pelleted, washed with 1 ml of sterile PBS, and resuspended in PBS to obtain ~1 × 10^9^ CFU/ml. One nanoliter of this bacterial suspension was microinjected into the bloodstream via the circulation valley with an Olympus SZ61 or SZX10 stereomicroscope together with a YOU-1 micromanipulator (Narishige), a Narishige IM-200 microinjector, and a JUN-AIR model 3 compressor. For each experiment, the average number of CFU per injection was determined by adding 10 1-nl drops to 1 ml of 0.7% NaCl, which was then serially diluted and plated on Luria-Bertani (LB) agar plates. Mock-infected controls were inoculated with 1 nl of sterile PBS. Following injection, embryos were removed from the agar and placed individually into wells of a 48-well plate (Nunc) containing E3 medium and incubated at 28.5°C. For lethality assays, death was defined as absence of heart contraction and blood flow.

### Mouse sepsis model.

Outbred female Swiss-Webster mice (Charles River) that were 5 to 6 weeks old were briefly anesthetized by isoflurane inhalation and injected subcutaneously in the nape of the neck with 10^8^ CFU of bacteria in 200 µl of warm, sterile PBS (24, 30, 100). After 6 or 12 h, mice were euthanized and the kidneys, livers, and spleens were collected and weighed.

### Microarray data accession number.

Complete microarray data have been deposited in the Gene Expression Omnibus database (http://www.ncbi.nlm.nih.gov/geo/) under accession number GSE79665.
