# Supplementary material for: Similarly Lethal Strains of Extraintestinal Pathogenic Escherichia coli Trigger Markedly Diverse Host Responses in a Zebrafish Model of Sepsis
Source: mSphere. 2016 Apr 20;1(2):e00062-16. doi: 10.1128/mSphere.00062-16 (PMC4894679; doi:10.1128/mSphere.00062-16)
Supplement: Figure S1 [file sph002162069sf4.pdf]

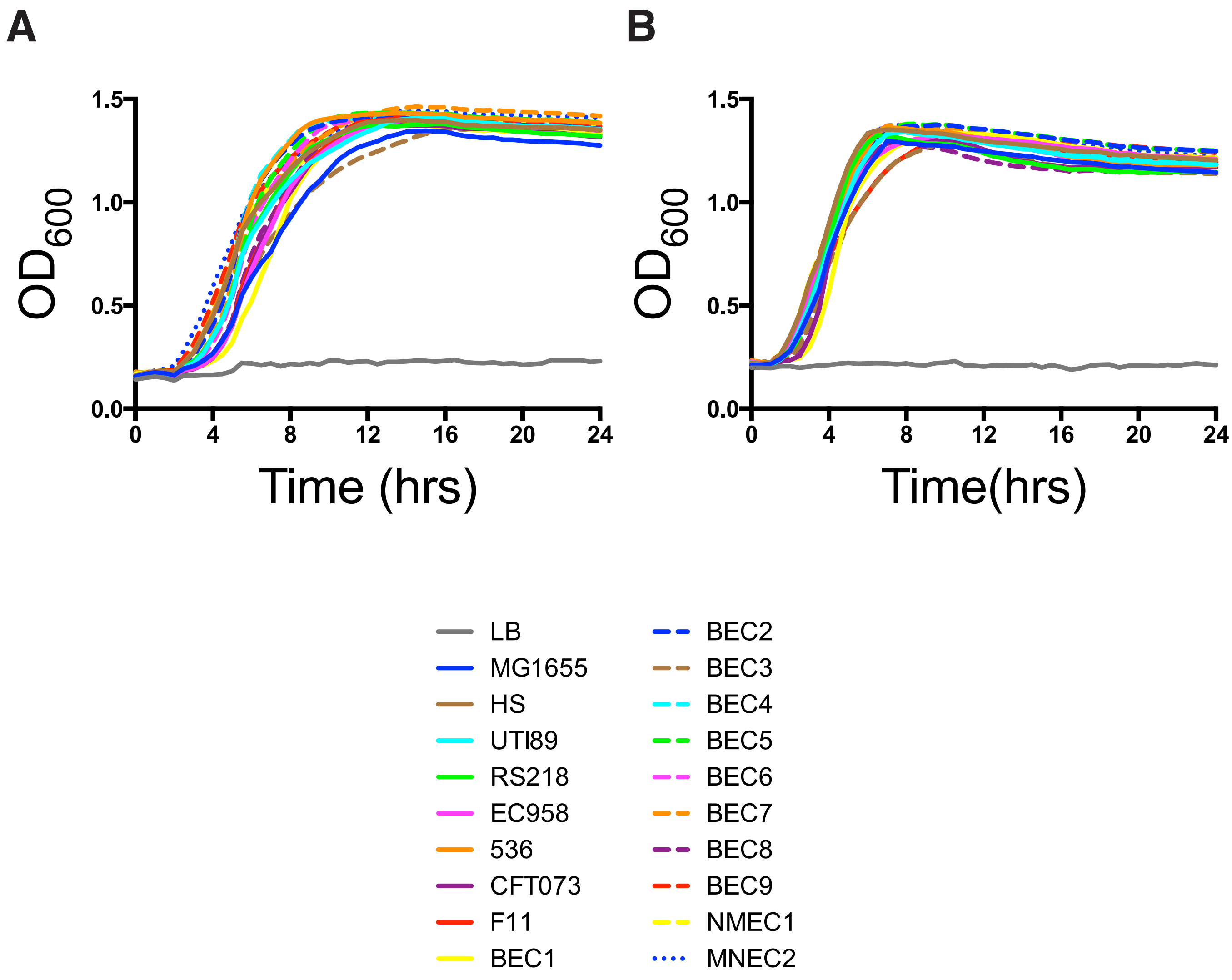

Supplemental Figure S1. Similar growth rates of *E. coli* strains in broth culture. (**A-B**) Representative data showing that all strains tested share similar growth kinetics in LB broth at 28.5°C (**A**) and 37°C (**B**). Each graph is representative of three independent experiments performed in quadruplicate.
