# Supplementary material for: Similarly Lethal Strains of Extraintestinal Pathogenic Escherichia coli Trigger Markedly Diverse Host Responses in a Zebrafish Model of Sepsis
Source: mSphere. 2016 Apr 20;1(2):e00062-16. doi: 10.1128/mSphere.00062-16 (PMC4894679; doi:10.1128/mSphere.00062-16)
Supplement: Figure S2 [file sph002162069sf5.pdf]

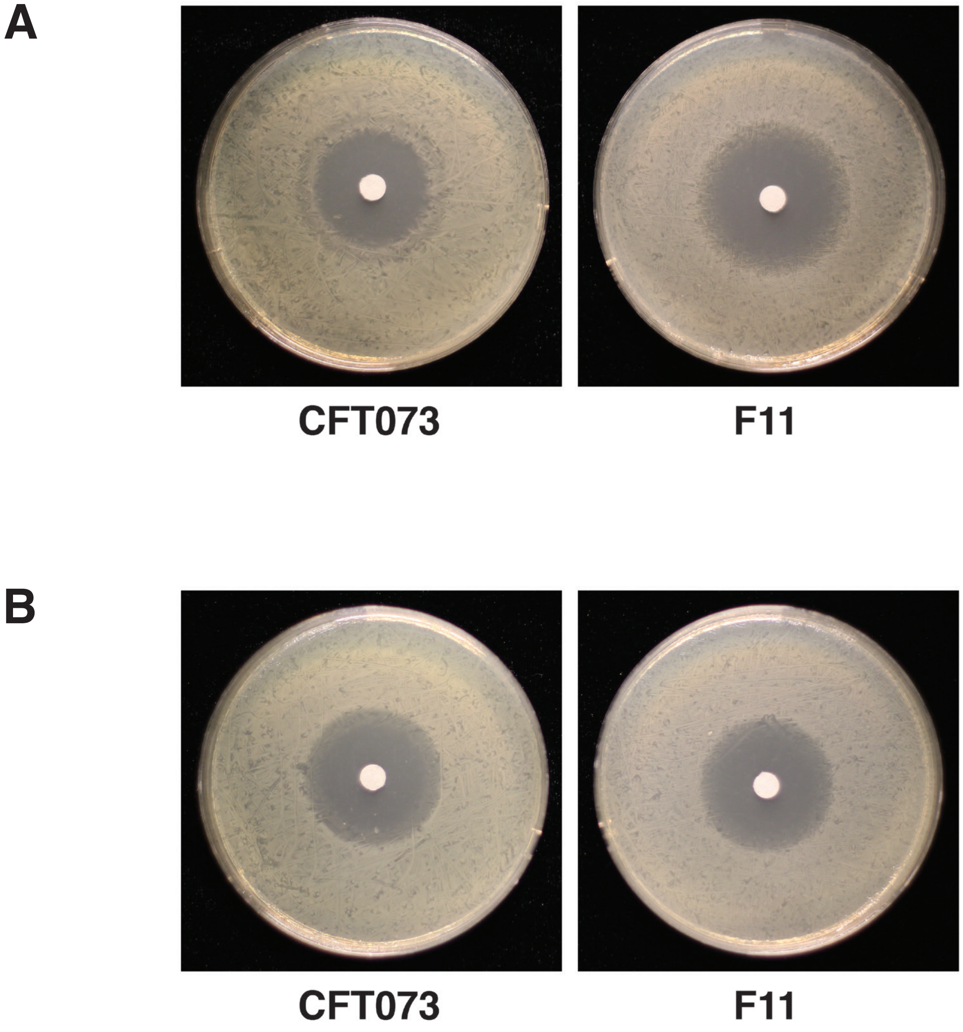

Supplemental Figure S2. CFT073 and F11 are similarly sensative to cipro loxacin *in vitro*.

(**A and B**) Representative images of ciprofloxacin disc diffusion assays on LB plates incubated overnight at 28.5°C (**A**) or 37°C (**B**). Each disc contains 5 µg ciprofloxacin.
