# Supplementary material for: Similarly Lethal Strains of Extraintestinal Pathogenic Escherichia coli Trigger Markedly Diverse Host Responses in a Zebrafish Model of Sepsis
Source: mSphere. 2016 Apr 20;1(2):e00062-16. doi: 10.1128/mSphere.00062-16 (PMC4894679; doi:10.1128/mSphere.00062-16)
Supplement: Figure S3 [file sph002162069sf6.pdf]

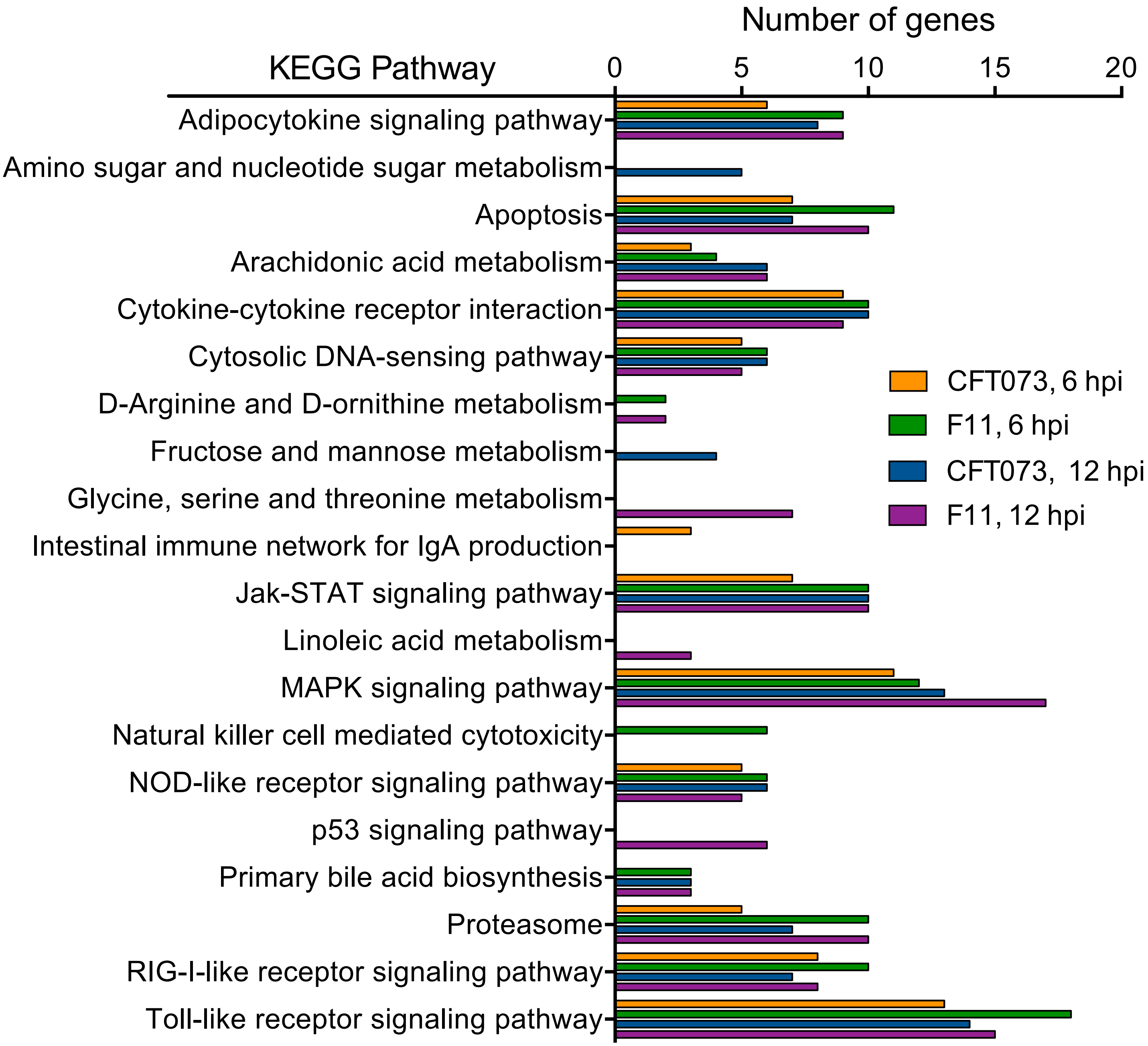

Supplemental Figure S3. CFT073 and F11 have differential effects on host biological pathways. Quantification of differentially expressed genes (fold change ≥ 2, $P \leq 0.05$) within KEGG pathways that are affected in response to systemic infections of zebrafish embryos with CFT073 and/or F11, as determined by DAVID.
