# Supplementary material for: Similarly Lethal Strains of Extraintestinal Pathogenic Escherichia coli Trigger Markedly Diverse Host Responses in a Zebrafish Model of Sepsis
Source: mSphere. 2016 Apr 20;1(2):e00062-16. doi: 10.1128/mSphere.00062-16 (PMC4894679; doi:10.1128/mSphere.00062-16)
Supplement: Figure S4 [file sph002162069sf7.pdf]

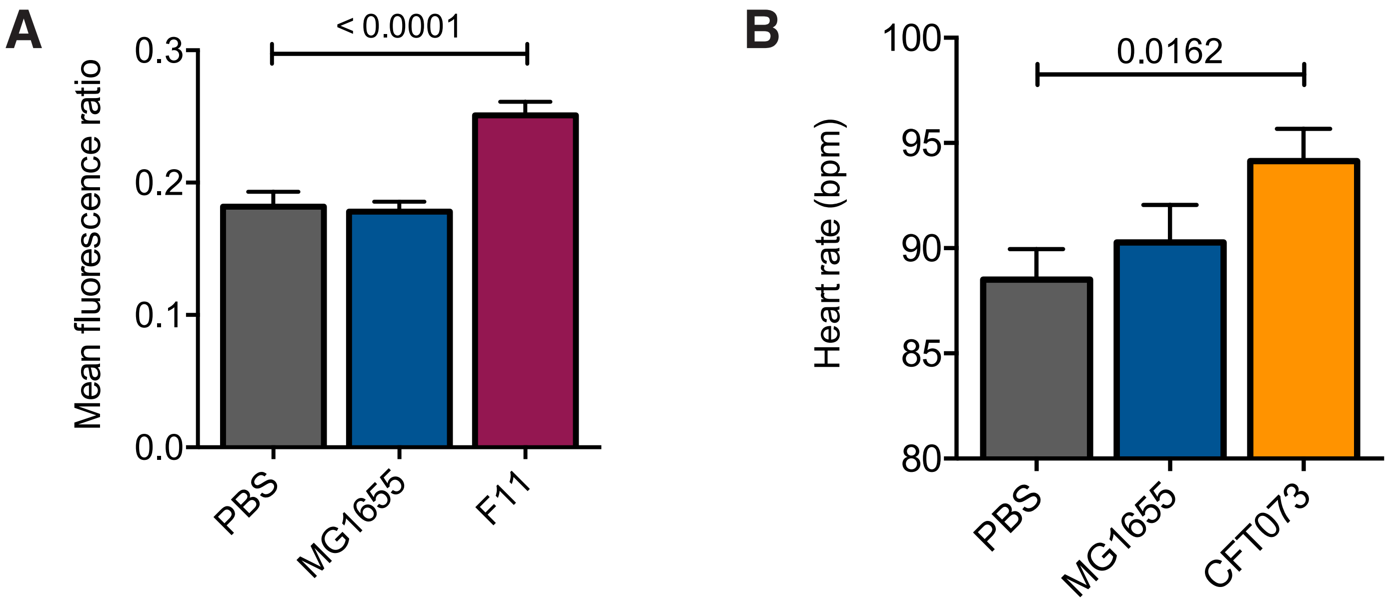

Supplemental Figure S4. ExPEC induces vascular leakage and tachycardia in zebrafish embryos.

(**A**) Endothelial leakage in infected and control PBS-injected embryos at 9 hpi. Dextran leakage was quantified by calculating the ratio of fluorescent intensity within the myotomes to the underlying vasculature. Data indicate mean values ± SEM from 2 independent experiments; n=9-15 embryos with 3 measurement sites used per fish.

(**B**) Heart rate (beats/min) in infected and PBS-injected control zebrafish embryos at 6 hpi. Each bar represents mean values ± SEM from 2 independent experiments; n=10-12 embryos. The indicated *P* values were determined by unpaired Student's t tests.

Additional information related to Supplemental Figure S4: Zebrafish embryos infected with lethal ExPEC isolates have pathologies like those seen in human sepsis.

Clinically, sepsis is diagnosed when there is a documented source of infection as well as at least two of the following symptoms: fever (temperature above 38°C) or hypothermia (temperature below 35.5°C), tachypnea (more than 20 breaths per minute), hypocapnia ($PaCO_2$ of less than 32 torr), tachycardia (more than 90 beats per minute), and ocytosis (more than 12,000/mm^3) or leukopenia (less than 4,000/mm^3) (11). Additional pathologies commonly seen during sepsis include endothelial dysfunction and consequent loss of barrier function leading to progressive subcutaneous edema and clotting abnormalities, including DIC. Considering these criteria, we set out to determine if zebrafish embryos infected with ExPEC display any overt signs of sepsis like those seen clinically in human patients.

Given that changes in body temperature are less applicable in ectothermic organisms such as zebrafish, we did not evaluate fever or hypothermia in ExPEC-infected embryos. ExPEC-induced leukopenia in zebrafish is suggested by previous work from our group in which we observed that F11- or CFT073-infected zebrafish have notably diminished numbers of phagocytes by 12 hpi, relative to MG1655-infected controls (12). As a measurement of vascular leakage, we injected fluorescent dextran (70 kDa) into the bloodstream of embryos and quantified its movement from the vasculature into host tissues (myotomes) after 15 min. In comparison with control embryos that were injected with non-pathogenic MG1655 or PBS, embryos that were infected with F11 had significantly higher amounts of vascular leakage by 9 hpi (**Supplemental Figure S4A**). These events could also contribute to the edema that

becomes manifest within the pericardial cavity and at other sites within ExPEC-infected embryos at later times points (see **Figs. 2A**, **3E**, and **3G**). Heart rate measurements showed that ExPEC can also induce tachycardia during systemic infections at 6 hpi (**Supplemental Figure S4B**), though heart rates eventually decline as the embryos succumb to the infection. Together these data demonstrate that infected zebrafish embryos display pathologies like those seen in human sepsis patients.
