# Supplementary material for: Similarly Lethal Strains of Extraintestinal Pathogenic Escherichia coli Trigger Markedly Diverse Host Responses in a Zebrafish Model of Sepsis
Source: mSphere. 2016 Apr 20;1(2):e00062-16. doi: 10.1128/mSphere.00062-16 (PMC4894679; doi:10.1128/mSphere.00062-16)
Supplement: Figure S5 [file sph002162069sf8.pdf]

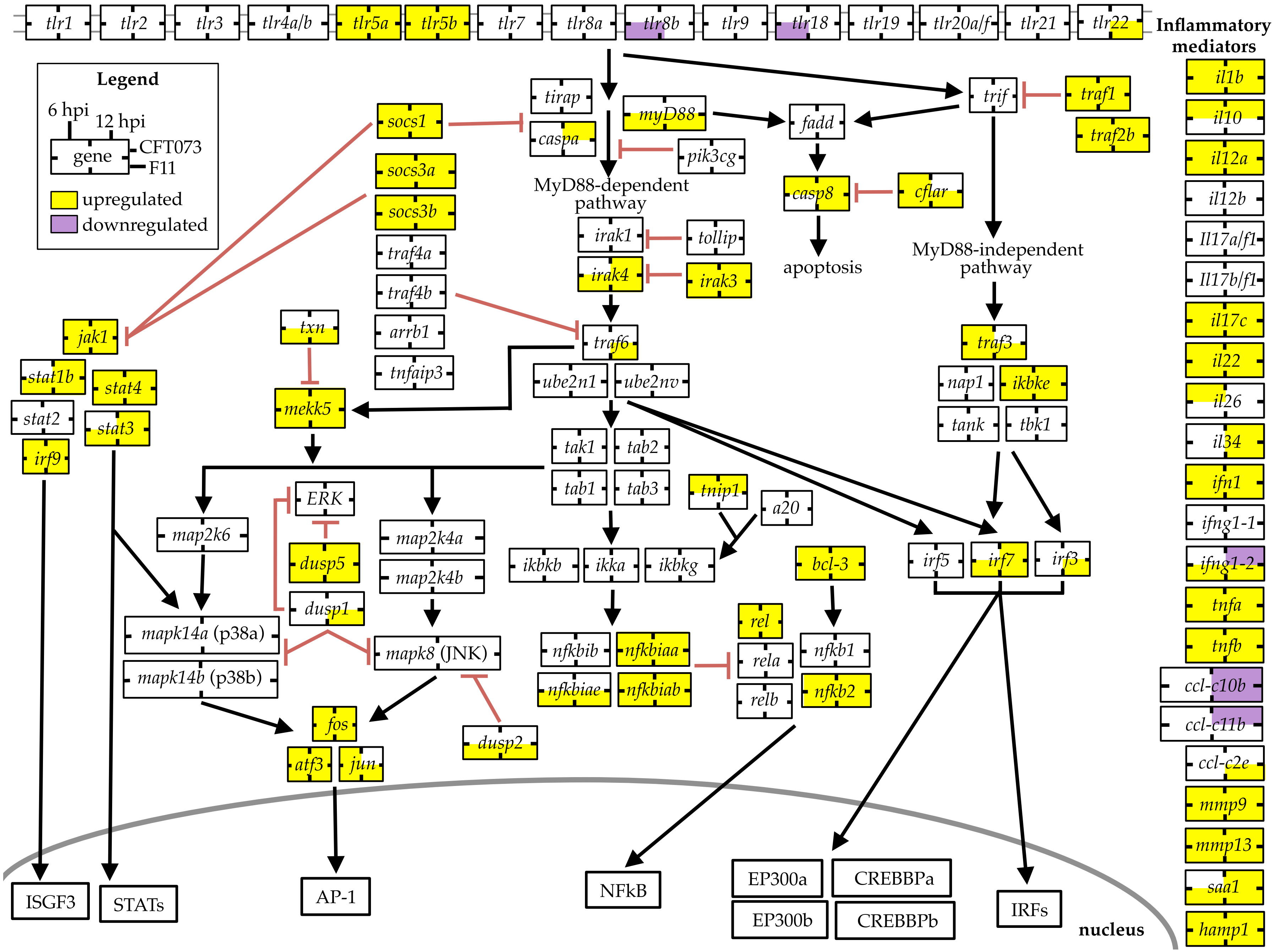

Supplemental Figure S5. CFT073 and F11 have overlapping, but distinct, effects on the expression of TLR pathway genes and downstream inflammatory mediators.
Diagram shows differentially expressed host genes at 6 and 12 hpi with CFT073 or F11. Upregulated genes are denoted in yellow, while those that are downregulated are purple (fold change up or down ≥ 2.0; P ≤ 0.05).
