# Supplementary material for: Similarly Lethal Strains of Extraintestinal Pathogenic Escherichia coli Trigger Markedly Diverse Host Responses in a Zebrafish Model of Sepsis
Source: mSphere. 2016 Apr 20;1(2):e00062-16. doi: 10.1128/mSphere.00062-16 (PMC4894679; doi:10.1128/mSphere.00062-16)
Supplement: Figure S6 [file sph002162069sf9.pdf]

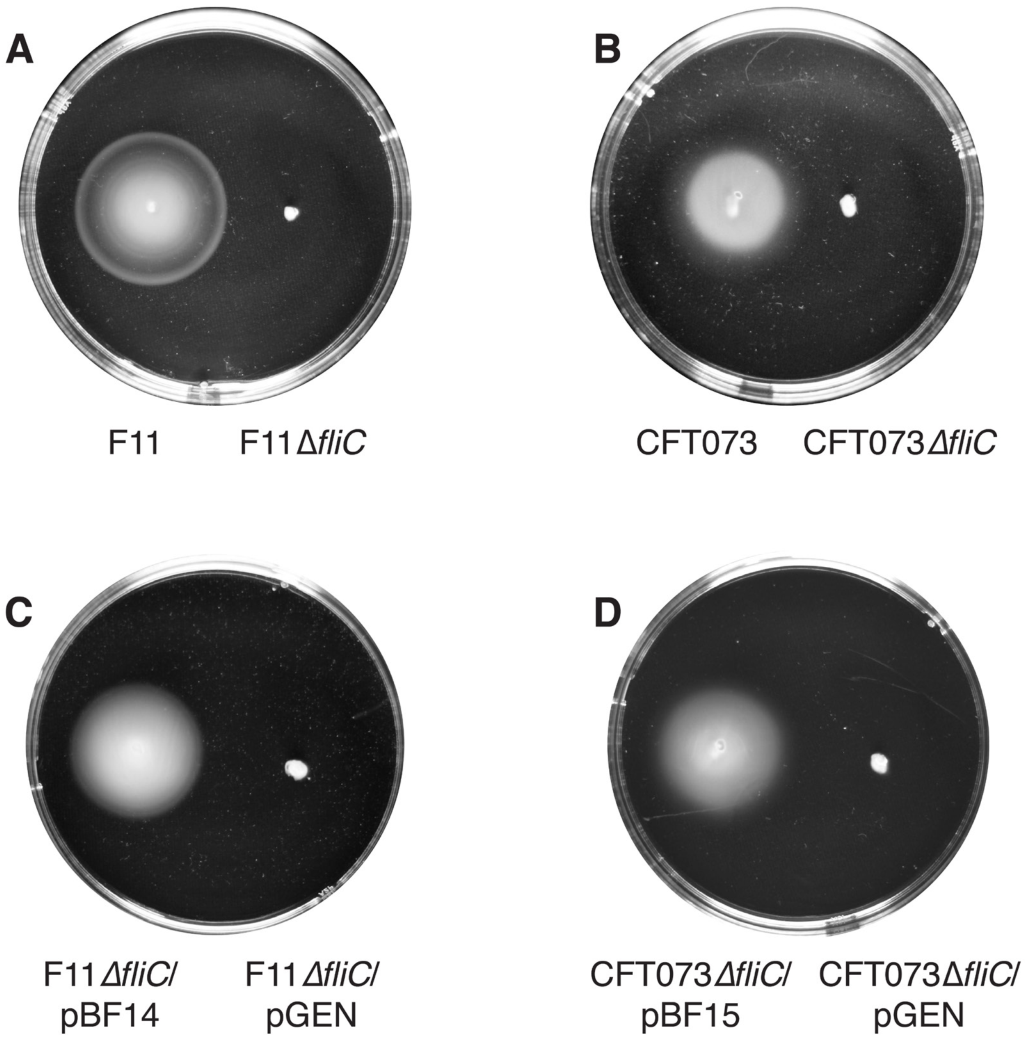

Supplemental Figure S6. Functional verification and complementation of *fliC* mutants.

(**A-B**) Deletion of *fliC* in F11 (**A**) and CFT073 (**B**) renders both pathogens immobile on 0.1% LB agar plates. Wild type strains are also pictured as controls.

(**C**) Complementation of F11Δ*fliC* with pBF14 restores motility on 0.1% LB agar plates, while the mutant carrying the empty vector pGEN-MCS (pGEN) remains immobile.

(**D**) Complementation of CFT073Δ*fliC* with pBF15 restores motility on 0.1% LB agar plates, while the mutant carrying the empty vector remains immobile.
