# Supplementary material for: Similarly Lethal Strains of Extraintestinal Pathogenic Escherichia coli Trigger Markedly Diverse Host Responses in a Zebrafish Model of Sepsis
Source: mSphere. 2016 Apr 20;1(2):e00062-16. doi: 10.1128/mSphere.00062-16 (PMC4894679; doi:10.1128/mSphere.00062-16)
Supplement: Table S1 [file sph002162069st1.pdf]

Supplemental Table S1. Bacterial Strains and Plasmids.

E. coli Strain/Plasmid	Description	Source or Reference
Strains		
F11	Cystitis isolate (O6:K2:H31)	(1)
CFT073	Urosepsis isolate (O6:K2:H1)	(2)
UTI89	Cystitis isolate (O18:K1:H7)	(3)
536	Pyelonephritis isolate (O6:K15:H31)	(4)
RS218	Neonatal meningitis isolate (O18ac:K1:H7)	(3, 5)
EC958	UTI isolate (O25b:H4)	(6)
HS	Commensal gut isolate (O9:H4)	(7)
MG1655	K12 lab strain	(8)
NMEC1	Human neonatal meningitis isolate	ARUP
NMEC2	Human neonatal meningitis isolate	ARUP
BEC1	Human blood isolate	ARUP
BEC2	Human blood isolate	ARUP
BEC3	Human blood isolate	Weyrich lab
BEC4	Human blood isolate	Weyrich lab
BEC5	Human blood isolate	H. Crandall
BEC6	Human blood isolate	H. Crandall
BEC7	Human blood isolate	H. Crandall
BEC8	Human blood isolate	H. Crandall
BEC9	Human blood isolate	H. Crandall
CFT073Δ*fliC*	CFT073 *fliC::kan*	This study
F11Δ*fliC*	F11 *fliC::kan*	This study
Plasmids		
p*fliC-lux*	Encodes P_{fliC} luciferase reporter, Amp^R	(9)
pKM208	Encodes IPTG inducible lambda Red recombinase, Amp^R	(10)
pGEN-MCS	High-retention plasmid containing multiple-cloning site, Amp^R	(9)
pBF14	Carries *fliC* with native promoter region from F11, Amp^R	This study
pBF15	Carries *fliC* with native promoter region from CFT073, Amp^R	This study
