# Supplementary material for: Similarly Lethal Strains of Extraintestinal Pathogenic Escherichia coli Trigger Markedly Diverse Host Responses in a Zebrafish Model of Sepsis
Source: mSphere. 2016 Apr 20;1(2):e00062-16. doi: 10.1128/mSphere.00062-16 (PMC4894679; doi:10.1128/mSphere.00062-16)
Supplement: Table S2 [file sph002162069st2.pdf]

Supplemental Table 2. Primers used in this study.

qRT-PCR	Sequence (5'-3')
**zEF1α**	
Forward	TGAGCGTGGTATCACCATTG
Reverse	CAACACCACCAGCAACAATC
**zIL1β**	
Forward	TGGACTTCGCAGCACAAAATG
Reverse	CACTTCACGCTCTTGGATGA
**zTNFα**	
Forward	AAGGAGAGTTGCCTTTACCG
Reverse	ATTGCCCTGGGTCTTATGG
**zSAA1**	
Forward	GGAACTATGAAGCTGCACAGCGG
Reverse	CCTCTGCGAATGAGACCTTG
**zHAMP**	
Forward	TGCAGGAGAACCAACATCTG
Reverse	AGCAGTATCCGCAGCCTTTA
**zMMP9**	
Forward	CATTAAAGATGCCCTGATGTATCCC
Reverse	AGTGGTGGTCCGTGGTTGAG
**zIL10**	
Forward	TCACGTCATGAACGAGATCC
Reverse	CCTCTTGCATTTCACCATATCC
**zIFN1**	
Forward	TCTGCGTCTACTTGCGAATG
Reverse	GGCTTGGAAATGGTGTCTCC
**zIL8**	
Forward	TGTGTTATTGTTTTCCTGGCATTTC
Reverse	GCGACAGCGTGGATCTACAG
**zIL17c**	
Forward	GTCGGAGAGCAAAGTGGAAG
Reverse	GCAGCCATCACACAAACACT
**zIL6**	
Forward	TCCTCAAACCTTCAGACCGC
Reverse	TCAGGACGCTGTAGATTCGC

Gene KO/confirmation plasmids	Sequence (5'-3')
**CFT073*Δflic* Knock Out**	
Forward	CGTAATCAACGACTTGCAATATAGGATAACGAATCTGTGTAGGCTGGAGCTGCTTCG
Reverse	GGCGTTGCCGTCAGTCTCAGTTAATCAGGTTACGGCGACATATGAATATCCTCCTTAG
**CFT073*Δflic* Confirmation**	
Forward	CGACAGACGATAACAGGGTTG
Reverse	ATCCGGCCTACAAAAATGTG

**F11*Δflic* Knock Out**	
Forward	CGTAATCAACGACTTGCAATATAGGATAACGAATCTGTGTAGGCTGG AGCTGCTTCG
Reverse	GGCGTTGCCGTCAGTCTCAGTTAATCAGGTTACAACGACATATGAATA TCCTCCTTAG
**F11*Δflic* Confirmation**	
Forward	CGACAGACGATAACAGGGTTG
Reverse	CTTACCCGGCCTACAAAATG
**pBF14/pBF15**	
Forward	GATCGAAGCTTGTTATCGGCCTGAATTGCGC
Reverse	GATTGCCATTGGTTAACCCTGCAGCAGAGACAG
**pBF14/pBF15 Confirmation**	
Forward	GCACTGTCTCTGTTATTCAGG
Reverse	CCAGCAGCAGTTACAAACTCAAG
