# Supplementary material for: Similarly Lethal Strains of Extraintestinal Pathogenic Escherichia coli Trigger Markedly Diverse Host Responses in a Zebrafish Model of Sepsis
Source: mSphere. 2016 Apr 20;1(2):e00062-16. doi: 10.1128/mSphere.00062-16 (PMC4894679; doi:10.1128/mSphere.00062-16)
Supplement: Text S1 [file sph002162069s10.pdf]

SUPPLEMENTAL MATERIALS AND METHODS

Ethics statement

Animals used in this study were handled in accordance with University of Utah and IACUC-approved protocols (Protocol number 10-02014) following standard guidelines described at www.zfin.org and in the Guide for the Care and Use of Laboratory Animals, 8th Edition.

Bacterial strains and plasmids

The bacterial strains and plasmids used in this study are listed in **Supplemental Table S1**. Bacteria used for infecting zebrafish and mice were grown statically at 37°C for 24 h in 12 ml modified M9 minimal medium (6 g/L Na_2HPO_4, 3 g/L KH_2PO_4, 1 g/L NH_4Cl, 0.5 g/L NaCL, 1 mM $MgSO_4$, 0.1 mM $CaCl_2$, 0.1% glucose, 0.0025% nicotinic acid, 0.2% casein amino acids, and 16.5 µg/mL thiamine in H_2O; pH 7.2). Strains carrying pGEN-GFP(LVA) or p*fliC*-lux were grown in medium containing ampicillin (50 mg/ml). Targeted deletion of *fliC* in F11 and CFT073 was performed using lambda Red-mediated recombination, as described previously (10). Deletion of the *fliC* in each strain was confirmed by PCR and by lack of motility on swim agar plates (**Supplemental Fig. S6**), as described in (13). FliC expression constructs pBF14 and pBF15 were made by cloning *fliC* plus 225 bp of upstream sequences from F11 or CFT073, respectively, into the high-retention, low copy number plasmid pGEN-MCS (9). Primers for the generation and confirmation of the strains and plasmids used in this study can be found in **Supplemental Table S2**.

Zebrafish embryos

Wild type AB zebrafish and the transgenic lines Tg(*krt8*:GFP) and Tg(*fli1a*:EGFP) were maintained as breeding colonies on a 14-h/10-h light/dark cycle. The Tg(*krt8*:GFP) fish express GFP in the outermost layer of epithelial cells while the Tg(*flia1*:eGFP) fish have eGFP-labeled endothelial cells and leukocytes (14, 15). Embryos were collected as mixed egg clutches and raised at 28.5°C in E3 medium (5 mM NaCl, 0.27 mM KCl, 0.4 mM $CaCl_2$, 0.16 mM $MgSO_4$; pH 7.4) containing 0.000016% methylene blue as an anti-fungal agent.

Enumeration of bacterial numbers in zebrafish embryos

Embryos were homogenized at the indicated time points in 500 µl of PBS containing 0.5% Triton X-100 using a mechanical PRO 250 homogenizer (PRO Scientific). Homogenates were serially diluted and plated on LB agar plates, which were then incubated overnight at 37°C.

Enumeration of bacterial numbers in mouse tissue

Excised tissues were homogenized in PBS containing 0.5% Triton X-100 using a Bullet Blender Storm 24 (Next Advance) in tubes containing 3.2 mm stainless steel beads. These beads disrupt host tissues, but do not affect bacterial viability. Homogenates were serially diluted and plated on LB agar plates for determination of bacterial titers.

Imaging of zebrafish embryos

To assess host cell viability, 12 hpi wild type zebrafish embryos were incubated for 30 min at 28.5°C in E3 medium with 10 μg/ml acridine orange. Samples were then washed several times with E3 medium, embedded in low melt agar, and imaged using a

fluorescent Olympus SZX10 stereomicroscope equipped with an Olympus DP72 camera. Transgenic Tg(*krt8*:GFP) and Tg(*fli1a*:GFP) embryos, as well as wild type embryos infected with F11/pGEN-GFP(LVA), were similarly imaged.

RNA isolation from zebrafish embryos

Pools of 15-20 embryos were manually homogenized in 1 ml QIAzol Lysis Reagent (Qiagen) and total RNA extracted using the Qiagen RNeasy Plus Universal Kit according to the manufacturer's instructions. Genomic DNA was removed by the gDNA Eliminator Solution (Qiagen). Samples used for microarray and qRT-PCR had a minimum RNA integrity number score of 9.

Microarray design, labeling, and hybridization

Microarray analysis was performed with biological quadruplicate samples using custom-designed 44k Agilent chips (16-18). Fluorescently labeled cRNA was synthesized using the Two-Color Low RNA Input Linear Amplification Kit (Agilent). Fluorescently labeled cRNA (825 ng) was then fragmented, combined with Hi-RPM Hybridization Buffer (Agilent), and hybridized using a SureHyb Hybridization chamber (Agilent). Slides were scanned in a G2505C Microarray Scanner (Agilent). Labeling, hybridizing, and scanning were performed by the Microarray and Genomic Analysis Core at the University of Utah.

Microarray data analysis

TIFF files of the scanned microarrays were processed using Feature Extraction Software version 10.5 (Agilent). Array data was then log2 transformed, Lowess-normalized, and median-centered. Fold changes were calculated by comparing infected

samples to mock-infected (PBS-injected) controls from the same clutch of embryos. Significance cutoffs were defined as an average of ≥ 2.0 fold change across all 4 arrays and $p \leq 0.05$. In order to restrict the false discovery rate, all *p*-values were adjusted using the Benjamini and Hochberg method (19). GO and KEGG analysis were performed using Database for Annotation, Visualization, and Integrated Discovery (DAVID) (http://david.abcc.ncifcrf.gov/) as described (20). Complete microarray data have been deposited in the Gene Expression Omnibus database (http://www.ncbi.nlm.nih.gov/geo/) under accession number GSE79665.

cDNA synthesis and qRT-PCR

cDNA was synthesized from 2 µg of RNA using the SuperScript III First-Strand Synthesis System (Invitrogen) in a 20 µl reaction volume. Following assembly of the master mix according to manufacturer instructions, samples were incubated at 25°C for 5 min, 55°C for 45 min, and 70°C for 15 min. Complimentary RNA was then removed by addition of RNase H (Invitrogen) for 15 min at 37°C.

qRT-PCR was performed on a LightCyler 480 instrument (Roche) following manufacturer recommendations. Cycling parameters were 95°C for 8 min to activate the polymerase followed by 40 cycles of 95°C for 4 sec, 60°C for 6 sec, and 72°C for 6 sec. Fluorescence measurements were taken at the end of each cycle. Melt curve analysis was performed to ensure that no primer dimers were amplified. All reactions were performed in technical duplicate. Sequences for the forward and reverse primers are listed in **Supplemental Table 2**. Results were normalized to transcript levels of the housekeeping gene *ef1*α (elongation factor 1-alpha) using $\Delta\Delta C_t$ analysis.

Quantification of mouse serum IL-6 levels

Blood was obtained from mice by cardiac puncture at the time of euthanasia. IL-6 concentrations in sera collected from the blood samples were determined by sandwich ELISA using clone MP5-20F3 as a capture antibody and clone MP5-2C11 as a detection antibody. Recombinant IL-6 was used as a standard. Antibodies for these assays were purchased from BD Pharmingen.

Isolation of flagella

Overnight bacterial cultures were back diluted 1:100 into 20 mL of tryptone broth and grown shaking (180 rpm) at 30°C until they reached mid-log phase. The presence of motile bacteria within the cultures was verified by microscopy prior to proceeding. Bacteria were pelleted by centrifugation at 6000 X g for 10 min at 4°C and then re-suspended in 3 mL sterile PBS. Bacterial suspensions were then passed between two 5 cc syringes connected with 0.28 mm I.D. polyethylene tubing (Becton Dickson) to shear flagella. Bacteria were then spun down at 6000 X g for 10 min at 4°C and supernatants containing sheared flagella were collected and stored at 4°C. Levels of FliC in the preparations were determined by Western blot using standard protocols (Abcam anti-Flagellin; 1:10,000) (21).

Quantification of flagella expression within infected zebrafish embryos

Zebrafish embryos were inoculated via the circulation valley with 2,000-2,500 CFU of CFT073/p*fliC*-lux or F11/p*fliC*-lux, as described above. A higher dose than the 1,000 CFU utilized in other assays was employed due to reduced fitness of bacteria carrying p*fliC*-lux, which contains a toxin-antitoxin-based plasmid retention system. Following

inoculation, embryos were distributed into wells containing E3 medium in a white-walled 96-well plate and incubated at 28.5°C. Luminescence was measured at 9 and 12 hpi using a BioTek Synergy H1 plate reader.

Quantification of TLR5 activation

TLR5 stimulation was assayed using HEK-Blue mTLR5 cells (Invivogen), a SEAP reporter line that expresses mouse TLR5. Briefly, cells were suspended at a concentration of 140,000 cells/mL in HEK-Blue detection media and 180 µL (~25,000 cells/well) of this suspension was placed into individual wells within a 96-well plate. Cells were then stimulated with 20 µL of the flagella preparations from CFT073 and F11 (each containing similar amounts of FliC as verified by Western blot analysis). Control samples were treated with PBS alone. After 15 h at 37°C, absorbance (640 nm) measurements were obtained using a BioTek Synergy H1 reader. For neutralizing antibody experiments, 1 µg/mL anti-mTLR5 or control IgG2a antibody (Invivogen) were added to cells and allowed to incubate for 1 h prior to addition of the flagella preparations.

Heart rate measurements in zebrafish embryos

Infected and PBS-injected control zebrafish embryos were briefly anesthetized with 0.77 tricaine (Sigma-Aldrich), embedded in low melt agarose (MO BIO Laboratories) without tricaine, and immersed in E3 media lacking methylene blue. After 1 h, embryos were filmed for ~1 min using an Olympus SZX10 stereomicroscope and an Olympus DP72 camera recording at 15 frames/sec. Heart rates (beats/min) were calculated from review of the footage.

Quantifying endothelial leakage in zebrafish embryos

Embryos were injected intravenously with 1 nL of tetramethylrhodamine-conjugated, lysine-fixable 70 kDa dextran (25 μg/ml; Life Technologies). After 15 min, embryos were fixed overnight at 4°C in PBS containing 4% paraformaldehyde. Samples were then washed 3X with PBS and taken through a series of washes with 30%, 50%, and 80% glycerol before mounting on slides using FluorSave Reagent (Calbiochem). Embryos were imaged using a fluorescent Olympus SZX10 stereomicroscope and an Olympus DP72 camera, ensuring equal exposure times for each sample. Using ImageJ (22), the mean fluorescent intensity for 3 specific myotomes, as well the lumen of the caudal artery, was measured (see Figure 9A). Endothelial leakage was defined as the ratio of the mean fluorescent intensity in a myotome to the mean fluorescent intensity in the lumen of the caudal artery below that myotome. This method accounts for variability in the amount of dextran injected.

Zebrafish ciprofloxacin assays

At the indicated time points, infected zebrafish embryos were transferred from E3 medium into E3 medium containing 50 µg/mL ciprofloxacin (pH 7.4). For enumeration of bacterial titers within ciprofloxacin-treated embryos, the fish were washed with E3 medium twice to remove ciprofloxacin and then homogenized and plated as described above.

Statistical analysis

Except where indicated, *P* values were calculated by two-tailed Student's *t* tests, Mann-Whitney U tests, or log-rank tests using Prism 6.0e software (GraphPad Software). Values of less than 0.05 were defined as significant for all experiments.

SUPPLEMENTAL REFERENCES

1. **Lloyd AL, Rasko DA, Mobley HLT.** 2007. Defining genomic islands and uropathogen-specific genes in uropathogenic *Escherichia coli*. J Bacteriol **189:**3532-3546.
2. **Welch RA, Burland V, Plunkett G, 3rd, Redford P, Roesch P, Rasko D, Buckles EL, Liou SR, Boutin A, Hackett J, Stroud D, Mayhew GF, Rose DJ, Zhou S, Schwartz DC, Perna NT, Mobley HL, Donnenberg MS, Blattner FR.** 2002. Extensive mosaic structure revealed by the complete genome sequence of uropathogenic *Escherichia coli*. Proc Natl Acad Sci U S A **99:**17020-17024.
3. **Mulvey MA, Schilling JD, Hultgren SJ.** 2001. Establishment of a persistent *Escherichia coli* reservoir during the acute phase of a bladder infection. Infect Immun **69:**4572-4579.
4. **Brzuszkiewicz E, Bruggemann H, Liesegang H, Emmerth M, Olschlager T, Nagy G, Albermann K, Wagner C, Buchrieser C, Emody L, Gottschalk G, Hacker J, Dobrindt U.** 2006. How to become a uropathogen: comparative genomic analysis of extraintestinal pathogenic *Escherichia coli* strains. Proc Natl Acad Sci U S A **103:**12879-12884.
5. **Xie Y, Kolisnychenko V, Paul-Satyaseela M, Elliott S, Parthasarathy G, Yao Y, Plunkett G, 3rd, Blattner FR, Kim KS.** 2006. Identification and characterization of *Escherichia coli* RS218-derived islands in the pathogenesis of *E. coli* meningitis. J Infect Dis **194:**358-364.
6. **Totsika M, Beatson SA, Sarkar S, Phan MD, Petty NK, Bachmann N, Szubert M, Sidjabat HE, Paterson DL, Upton M, Schembri MA.** 2011. Insights into a multidrug resistant *Escherichia coli* pathogen of the globally disseminated ST131 lineage: genome analysis and virulence mechanisms. PLoS ONE **6:**e26578.
7. **Levine MM, Bergquist EJ, Nalin DR, Waterman DH, Hornick RB, Young CR, Sotman S.** 1978. *Escherichia coli* strains that cause diarrhoea but do not produce heat-labile or heat-stable enterotoxins and are non-invasive. Lancet **1:**1119-1122.
8. **Blattner FR, Plunkett G, 3rd, Bloch CA, Perna NT, Burland V, Riley M, Collado-Vides J, Glasner JD, Rode CK, Mayhew GF, Gregor J, Davis NW, Kirkpatrick HA, Goeden MA, Rose DJ, Mau B, Shao Y.** 1997. The complete genome sequence of *Escherichia coli* K-12. Science **277:**1453-1462.
9. **Lane MC, Alteri CJ, Smith SN, Mobley HL.** 2007. Expression of flagella is coincident with uropathogenic *Escherichia coli* ascension to the upper urinary tract. Proc Natl Acad Sci U S A **104:**16669-16674.

10. **Murphy KC, Campellone KG.** 2003. Lambda Red-mediated recombinogenic engineering of enterohemorrhagic and enteropathogenic *E. coli*. BMC Mol Biol **4:**11.
11. **Dellinger RP, Levy MM, Rhodes A, Annane D, Gerlach H, Opal SM, Sevransky JE, Sprung CL, Douglas IS, Jaeschke R, Osborn TM, Nunnally ME, Townsend SR, Reinhart K, Kleinpell RM, Angus DC, Deutschman CS, Machado FR, Rubenfeld GD, Webb SA, Beale RJ, Vincent J-L, Moreno R.** 2013. Surviving Sepsis Campaign. Critical Care Medicine **41:**580-637.
12. **Wiles TJ, Bower JM, Redd MJ, Mulvey MA.** 2009. Use of zebrafish to probe the divergent virulence potentials and toxin requirements of extraintestinal pathogenic *Escherichia coli*. PLoS Path **5:**e1000697.
13. **Kulesus RR, Diaz-Perez K, Slechta ES, Eto DS, Mulvey MA.** 2008. Impact of the RNA chaperone Hfq on the fitness and virulence potential of uropathogenic *Escherichia coli*. Infect Immun **76:**3019-3026.
14. **Gong Z, Ju B, Wang X, He J, Wan H, Sudha PM, Yan T.** 2002. Green fluorescent protein expression in germ-line transmitted transgenic zebrafish under a stratified epithelial promoter from *keratin8*. Dev Dynamics **223:**204-215.
15. **Lawson ND, Weinstein BM.** 2002. In Vivo Imaging of Embryonic Vascular Development Using Transgenic Zebrafish. Developmental Biology **248:**307-318.
16. **Stockhammer OW, Zakrzewska A, Hegedus Z, Spaink HP, Meijer AH.** 2009. Transcriptome profiling and functional analyses of the zebrafish embryonic innate immune response to Salmonella infection. J Immunol**182:**5641-5653.
17. **van der Sar AM, Spaink HP, Zakrzewska A, Bitter W, Meijer AH.** 2009. Specificity of the zebrafish host transcriptome response to acute and chronic mycobacterial infection and the role of innate and adaptive immune components. Mol Immunol **46:**2317-2332.
18. **Stockhammer OW, Rauwerda H, Wittink FR, Breit TM, Meijer AH, Spaink HP.** 2010. Transcriptome analysis of Traf6 function in the innate immune response of zebrafish embryos. Mol Immunol **48:**179-190.
19. **Benjamini Y, Hochberg Y.** 1995. Controlling the false discovery rate: a practical and powerful approach to multiple testing. J Royal Stat Soc, Series B **57:**289-300.
20. **Huang da W, Sherman BT, Lempicki RA.** 2009. Systematic and integrative analysis of large gene lists using DAVID bioinformatics resources. Nat Protoc **4:**44-57.
21. **Eto DS, Jones TA, Sundsbak JL, Mulvey MA.** 2007. Integrin-mediated host cell invasion by type 1-piliated uropathogenic *Escherichia coli*. PLoS Path **3:**e100.
22. **Schneider CA, Rasband WS, Eliceiri KW.** 2012. NIH Image to ImageJ: 25 years of image analysis. Nat Methods **9:**671-675.
